# On-demand synthesis of high-quality, blue-light-active ZnSe colloidal quantum wires

**DOI:** 10.1093/nsr/nwac025

**Published:** 2022-02-26

**Authors:** Yi Li, Chong Zhang, Jie Tian, Liang Wu, Guo-Qiang Liu, Hui-Hui Li, Yu-Zhuo Zhang, Zhen-Chao Shao, Zhen He, Shu-Hong Yu

**Affiliations:** Department of Chemistry, Institute of Biomimetic Materials and Chemistry, Anhui Engineering Laboratory of Biomimetic Materials, Division of Nanomaterials and Chemistry, Hefei National Research Center for Physical Sciences at the Microscale, University of Science and Technology of China, Hefei 230026, China; Department of Chemistry, Institute of Biomimetic Materials and Chemistry, Anhui Engineering Laboratory of Biomimetic Materials, Division of Nanomaterials and Chemistry, Hefei National Research Center for Physical Sciences at the Microscale, University of Science and Technology of China, Hefei 230026, China; Engineering and Materials Science Experiment Center, University of Science and Technology of China, Hefei 230026, China; Department of Chemistry, Institute of Biomimetic Materials and Chemistry, Anhui Engineering Laboratory of Biomimetic Materials, Division of Nanomaterials and Chemistry, Hefei National Research Center for Physical Sciences at the Microscale, University of Science and Technology of China, Hefei 230026, China; Department of Chemistry, Institute of Biomimetic Materials and Chemistry, Anhui Engineering Laboratory of Biomimetic Materials, Division of Nanomaterials and Chemistry, Hefei National Research Center for Physical Sciences at the Microscale, University of Science and Technology of China, Hefei 230026, China; Department of Chemistry, Institute of Biomimetic Materials and Chemistry, Anhui Engineering Laboratory of Biomimetic Materials, Division of Nanomaterials and Chemistry, Hefei National Research Center for Physical Sciences at the Microscale, University of Science and Technology of China, Hefei 230026, China; Department of Chemistry, Institute of Biomimetic Materials and Chemistry, Anhui Engineering Laboratory of Biomimetic Materials, Division of Nanomaterials and Chemistry, Hefei National Research Center for Physical Sciences at the Microscale, University of Science and Technology of China, Hefei 230026, China; Department of Chemistry, Institute of Biomimetic Materials and Chemistry, Anhui Engineering Laboratory of Biomimetic Materials, Division of Nanomaterials and Chemistry, Hefei National Research Center for Physical Sciences at the Microscale, University of Science and Technology of China, Hefei 230026, China; Department of Chemistry, Institute of Biomimetic Materials and Chemistry, Anhui Engineering Laboratory of Biomimetic Materials, Division of Nanomaterials and Chemistry, Hefei National Research Center for Physical Sciences at the Microscale, University of Science and Technology of China, Hefei 230026, China; Department of Chemistry, Institute of Biomimetic Materials and Chemistry, Anhui Engineering Laboratory of Biomimetic Materials, Division of Nanomaterials and Chemistry, Hefei National Research Center for Physical Sciences at the Microscale, University of Science and Technology of China, Hefei 230026, China

**Keywords:** zinc selenide, colloidal synthesis, quantum wires, heavy-metal-free, solar conversion

## Abstract

Beyond the state-of-the-art Cd-containing quantum wires (QWs), heavy-metal-free semiconductor QWs, such as ZnSe, are of great interest for next-generation environmental-benign applications. Unfortunately, simultaneous, on-demand manipulation of their radial and axial sizes—that allows strong quantum confinement in the blue-light region—has so far been challenging. Here we present a two-step catalyzed growth strategy that enables independent, high-precision and wide-range controls over the diameter and length of ZnSe QWs. We find that a new epitaxial orientation between the cubic-phase Ag_2_Se solid catalyst and wurtzite ZnSe QWs kinetically favors the formation of defect-free ultrathin QWs. Thanks to their high uniformity, the resulting blue-light-active, phase-pure ZnSe QWs exhibit well-defined excitonic absorption with the 1S_e_–1S_h_ transition linewidth as narrow as sub-13 nm. Combining the transient absorption spectroscopy, we further show that surface electron traps in these ZnSe QWs can be eliminated by thiol passivation, which results in long-lived charge carriers and high-efficiency solar-to-hydrogen conversion.

## Teaser

A two-step catalytic growth strategy is reported to enable high-quality ZnSe quantum wires with precise and wide-range size control, which present exciting potentials for blue-light-driven photochemistry.

## INTRODUCTION

More than three decades of research has powered colloidal quantum dots as one of the utmost compelling materials for applications in light-emitting devices, lasers, photovoltaics, solar-to-fuel conversion and bioimaging [1–3]. As compared with those zero-dimensional quantum materials, their one-dimensional (1D) counterparts, i.e. quantum wires (QWs), are emerging alternatives—something that unites the benefits of both quantum dots and bulk materials [4]. Radially, the quantum confinement enables diameter-tailored band gaps and strong excitonic effects; axially, the bulk-like feature translates into enhanced optical absorption, feasible carrier transport [5] and low Auger recombination rates for carrier multiplication and lasing [6,7]. In addition, the 1D anisotropy enables QWs as versatile platforms for constructing functional heterostructures [8].

Despite their well-established synthetic methodologies and controllable optical properties, Cd-containing II–VI quantum materials have been impeded for next-generation environmental-benign applications, which in fact require stable, efficient, heavy-metal-free compositions [9–12]. ZnSe, a II–VI semiconductor with a direct band gap of ∼2.7 eV in bulk, represents one of the best potential candidates for blue-light-region applications, due to its relatively large exciton Bohr radius (∼3.8 nm) and the highest carrier mobility among II–VI chalcogenides [13,14]. In this context, with precise modulation on their size and shape, ZnSe QWs would hold great promise for polarized blue-light emission, detection, as well as blue-light-driven solar-to-fuel conversion [2,10,15–18].

Unfortunately, the colloidal synthesis of ZnSe QWs has been challenging in contrast to the benchmark Cd-based semiconductors. To date, synthetic strategies for ZnSe QWs mainly include anisotropic-controlled growth [19,20], oriented attachment [21–23] and solution–liquid/solid–solid catalyzed growth [24–29]. The anisotropic-controlled growth only produces tiny anisotropic ZnSe quantum rods with small aspect ratios. Contrarily, the oriented attachment approach usually yields ultralong QWs with diameters of sub-2 nm, as restricted by their starting materials of magic-sized clusters. Despite that further heating and ripening can transform the ultrathin QWs to shorter and slightly thicker quantum rods, their radial and axial size evolutions are negatively correlated [30–32]. Catalyzed growth can, in principle, allow independent control over the diameter and length of QWs [13,29]; however, it is now technically limited to ultralong, thick ZnSe nanowires beyond the quantum-confined regime.

Therefore, ZnSe nanowires produced from these previous synthetic strategies are substantially limited to the strong quantum confinement regime with near-violet-light absorption or to the bulk regime with undiscernible exciton features (Supplementary Table 1). Thus far, simultaneously, on demand manipulating the radial and axial sizes of ZnSe QWs at high precision that enables tunable, well-defined exciton features in the blue-light region remains both synthetically and technically challenging, which further impedes their blue-light-involved applications and our understanding toward the dimension-dependent excitonic behaviors.

Here, we achieve the on-demand synthesis of high-quality, blue-light-active ZnSe QWs by developing a flexible synthetic approach that permits independent manipulations on the diameter and length of ZnSe QWs. In this way, we bridge the gap between prior magic-sized ZnSe QWs and bulk-like ZnSe nanowires. As a proof-of-concept, we regulated the diameter of QWs from sub-5 nm of strong confinement to levels above the exciton Bohr diameter; the length of QWs ranges from tens of nanometers to several micrometers. We revealed that these wurtzite ZnSe QWs epitaxially grow along a unique crystalline direction that circumvents the ubiquitous stacking faults in conventional II–VI nanowires. This phase purity, in combination with the strong quantum confinement and high-precision size control, gives rise to ultranarrow 1S_e_–1S_h_ transition line widths. Such high-quality QWs display long-lived excited states after passivating their surface electron traps with thiol ligands, consequently exhibiting superior blue-light-driven solar-to-hydrogen conversion efficiency. Therefore, the high-quality, flexibly tunable ZnSe QWs presented here signify great technical advances in the colloidal synthesis field and will be of wide interest for blue-light-related optoelectronics and chemical reactions.

## RESULTS AND DISCUSSION

### On-demand synthesis of ZnSe QWs

**Figure 1.**
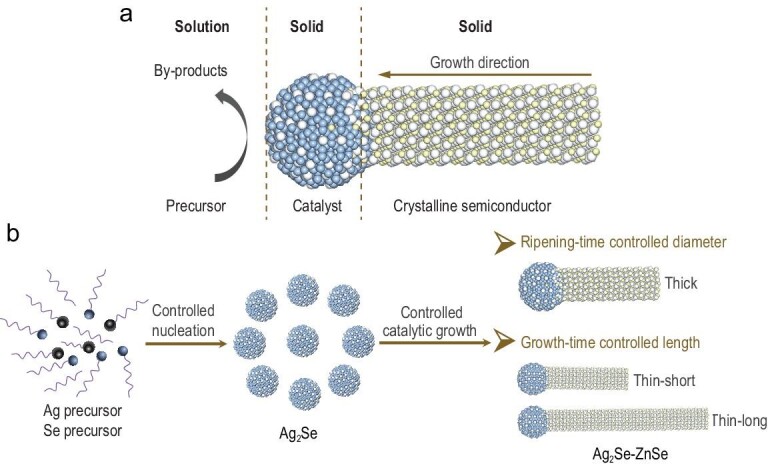
Schematic synthetic control over the radial and axial sizes of semiconductor nanowires. (a) Schematic solution–solid–solid growth mechanism of semiconductor nanowires. (b) Simultaneous radial and axial size control of ZnSe QWs achieved by independently tuning the ripening time and growth time.

For catalyzed growth in flask-based solution chemistry (Fig. 1a), semiconductor nanowires grow at the catalyst–semiconductor interface in a layer-by-layer manner. It thus offers an effective route to independently manipulate the radial and axial sizes of ZnSe QWs by finely tuning the size of catalysts and the growth time of QWs [29,33]. To grow diameter-controlled ultrathin QWs, small catalysts are required. Nevertheless, in conventional solution–liquid–solid growth, small liquid catalysts (e.g. Bi) tend to aggregate or coalesce into large ones, inhibiting the diameter control of QWs. This can be mitigated in solution–solid–solid growth by using superionic Ag_2_Se as solid catalysts, wherein foreign cations are incorporated into these superionic conductors via high-density cation vacancies.

To grow uniform, straight, high-quality ZnSe QWs, we took a two-step catalyzed growth methodology (Fig. 1b): first, synthesizing and purifying monodispersed small Ag_2_Se catalyst seeds; second, growing ZnSe QWs of varied diameters by controlling the ripening time of catalysts in solution before introducing the cation precursor. Notably, in the latter step, we keep the concentration of the anion precursor at a high level, which was reported to favor high-quality nanowires during the catalyzed growth [13,34]. This two-step growth is important for diameter control; otherwise, the catalysts will keep growing as the reaction proceeds, leading to non-uniform QWs in the end. We then control the QW length via growth time and the ratio of cation precursor to seeds.

**Figure 2.**
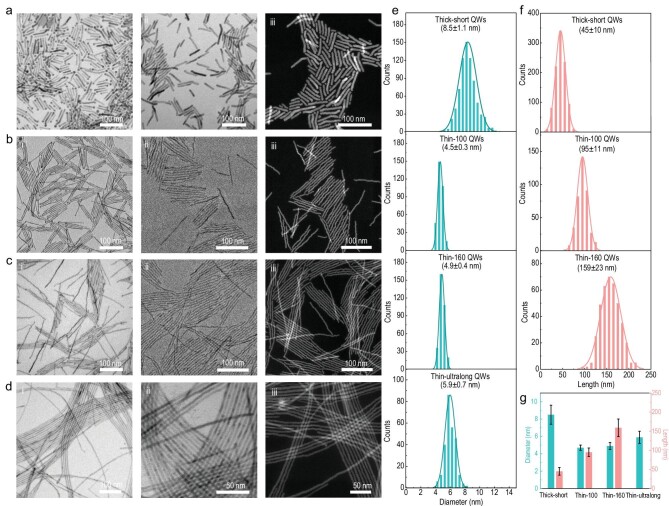
Synthetic control over the radial and axial sizes of ZnSe QWs. (a)–(d) TEM and HADDF-STEM images of ZnSe QWs with different sizes before (i) and after Ag_2_Se tip removal (ii, iii). (a) Thick-short ZnSe QWs. (b) Thin-100 ZnSe QWs. (c) Thin-160 ZnSe QWs. (d) Thin-ultralong ZnSe QWs. (e) and (f) Statistical diameter (e) and length (f) distributions of different QWs in (a)–(d). (g) Histograms of the average diameter and length of different ZnSe QWs. Error bars in (g) correspond to the standard deviation values of QW diameter (cyan) and length (pink).

Monodispersed Ag_2_Se nanoparticles with different diameters can be synthesized in oleylamine by controlling the reaction temperature (Supplementary Fig. 1; see experimental methods for synthetic details). Illustratively, we implemented Ag_2_Se nanoparticles with an average diameter of 5.4 ± 0.5 nm as the solid catalysts. Keeping these Ag_2_Se seeds ripening in the Se precursor solution before introducing the Zn precursor results in relatively thick, short quantum rods with Ag_2_Se tips of higher contrast at the end (Fig. 2a-i). Nevertheless, using Ag_2_Se seeds without purification yields inhomogeneous QWs with broader size distributions as a result of the uncontrolled ripening of the catalysts (Supplementary Fig. 2).

To obtain ultrathin ZnSe QWs, we injected the Ag_2_Se stock solution at the growth temperature and then added Zn precursor immediately to avoid ripening (Fig. 2b-i). We highlight that the high-concentration Se precursor is of central importance for high-quality QWs, whereas the low Se/Zn stoichiometry disables the catalyzed growth (Supplementary Fig. 3). We then examined the influences of different Zn precursors on the morphology and quality of resultant ZnSe QWs and found that zinc acetate yields the best QWs (Supplementary Fig. 4). By controlling the reaction time, we can flexibly and accurately tune the QW length (Supplementary Fig. 5). By keeping different amounts of Ag_2_Se seeds ripening at high temperature before the addition of the Zn precurosor, we can further finely modulate the Ag_2_Se catalyst diameters and thereby the ZnSe QW diameters (Supplementary Fig. 6). Such diameter control can take effects even with the simultaneous introduction of the Zn precursor—just by tuning the number of Ag_2_Se seeds—the lower the number of seeds, the higher the degree of ripening (Fig. 2c-i and Supplementary Fig. 7). Intriguingly, when replacing the Se precursor with SeO_2_, which subsequently forms Se-oleylamine complexes in solution and thus increases the ripening time of seeds, we have access to slightly thicker QWs with length reaching several micrometers (Fig. 2d-i and Supplementary Fig. 8).

To substantiate the high reproducibility of our synthetic method, we provide the low-magnification transmission electron microscopy (TEM) images of different ZnSe QWs (Supplementary Fig. 9), which are highly uniform. In fact, this method is of high flexibility in tailoring the quantum confinement effect of QWs by diameter modulations, which can span from ∼4 nm to scales approaching the bulk limit (Supplementary Fig. 10). We can also synthesize thicker ZnSe nanorods beyond the quantum-confined regime by using larger Ag_2_Se seeds obtained at higher temperature (Supplementary Fig. 11). Such high-level control of these blue-light active ZnSe QWs implies their great potentials in wavelength-specific/tunable optoelectronic applications, such as blue-light emission and detection.

Taking the thick-short Ag_2_Se–ZnSe as an example, we show that the Ag_2_Se nanoparticle selectively locates at one tip of the ZnSe nanowire thanks to the catalyzed growth strategy (Supplementary Fig. 12). According to Pearson's theory of hard and soft acids and bases, which is widely adopted in cation exchange reactions, we further removed the Ag_2_Se tips from these QWs with alkylphosphine—due to the fact that as compared to hard acid (Zn^2+^), the soft acid (Ag^+^) can selectively combine with the soft base (alkylphosphine) and then be extrated from nanowires. This ultimatelty outputs highly uniform, monodispersed plain ZnSe QWs with different sizes, as shown in the TEM images (Fig. 2a-ii to d-ii) [35]. The homogeneous contrast in the high-angle annular dark field scanning TEM images (Fig. 2a-iii to d-iii), in combination with quantitative analyses (Supplementary Fig. 13 and Supplementary Table 2) from energy-dispersive X-ray spectroscopy (EDS) and inductively coupled plasma atomic emission spectroscopy (ICP-AES), confirms that all Ag_2_Se tips were completely removed from QWs. The resultant ZnSe QWs contain almost stoichiometric Zn and Se (Supplementary Table 3) with an undetectable amount of Ag, which ensures that their electronic structures are unaffected. All QWs present extremely narrow radial and axial size distributions that locate in the quantum-confined regime (Fig. 2e and f). The average diameter and length of these QWs are in sequence (8.5 ± 1.1) × (45 ± 10), (4.5 ± 0.3) × (95 ± 11), (4.9 ± 0.4) × (159 ± 23) and (5.9 ± 0.7) × several micrometers (Fig. 2g). We thus note these four typical QWs respectively as thick-short, thin-100, thin-160 and thin-ultralong ones in the following description. The synthetic parameters for ZnSe QWs of different sizes are shown in Supplementary Table 4.

### Spectroscopic analysis

**Figure 3.**
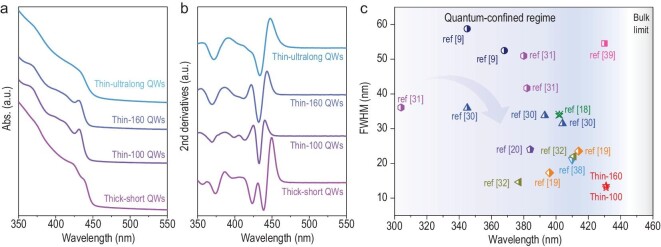
Optical characterization of ZnSe QWs. (a) Optical absorption spectra and (b) corresponding second derivatives of ZnSe QWs with varied sizes, which show well-defined excitonic features. (c) Plot of FWHM versus wavelength for the 1S_e_–1S_h_ exciton absorption of different ZnSe QWs.

To quantitatively examine the quantum confinement effect and monodispersity, we collected the optical absorption spectra of these ZnSe QWs (Fig. 3a). As compared with the band-edge absorption of the bulk ZnSe (∼2.7 eV), all four kinds of QWs exhibited pronounced, discrete excitonic transitions with obviously blue-shifted 1S_e_–1S_h_ first excitonic features—a consequence of quantum confinement. The well-defined exciton features enable us to ascribe them to different interband optical transitions (Supplementary Fig. 14). As far as we know, these are the best reported QWs with clear, narrow exciton transition features in the blue-light region (>420 nm).

Due to their anisotropy, 1D QWs present an interesting dimensionality-dependent excitonic behavior. To evaluate the dimensionality effect on the optical absorption of ZnSe QWs, we further present the second derivatives of their absorption spectra (Fig. 3b), wherein the excitonic transitions are well resolved. Among them, the thin-100 and thin-160 QWs show the sharpest 1S_e_–1S_h_ first excitonic features at ∼430 nm; the latter displays slightly red-shifted absorption, which we attribute to its marginally larger diameter and thus relatively weaker quantum confinement. In contrast to these thin QWs, the absorption of the thick-short QWs with diameter approaching the exciton Bohr diameter is obviously red-shifted due to the largely relaxed quantum confinement. The thin-ultralong QWs with diameters between ultrathin and thick ones present ground–state transition energy close to the ultrathin ones due to their similar radial sizes, while long QWs show an almost featureless extinction spectrum, which may result from interactions between quantum confinement and dielectric contrast as well as optical scattering in such 1D ultralong nanowires [6,36]. In comparison with these 1D QWs, the zero-dimensional ZnSe quantum dots with average diameter of 5.6 ± 0.6 nm show a very blue-shifted absorption locating at ∼423 nm—a result that correlates with the reduced dielectric contrast in the 1D system [37]. As such, these blue-light-active exciton absorption features are highly tunable by precisely modulating the radial and axial sizes of the ZnSe QWs.

To further validate the high quality and high uniformity of the synthetic ZnSe QWs, we then compared with prior reports on the full width at half maximum (FWHM) of the 1S_e_–S_h_ exciton absorption as fitted with a Gaussian function (Supplementary Fig. 15). Note that we compared their absorption FWHM instead of the emission FWHM, owing to the fact that the former is inherently related to nanowire sizes and therefore the band gaps of quantum-confined nanomaterials, whereas the latter is usually broadened by surface and crystalline defects. As shown in Fig. 3c and Supplementary Table 1, the ZnSe QWs presented here exhibit the narrowest FWHM of sub-13 nm in the blue-light region [9,18–20,30–32,38,39]. Such high-level size control together with the formation of the 1D exciton offer a path for polarized light emission, photovoltaics and solar fuel production in the blue-light region.

### Crystal structure analysis and growth mechanism

**Figure 4.**
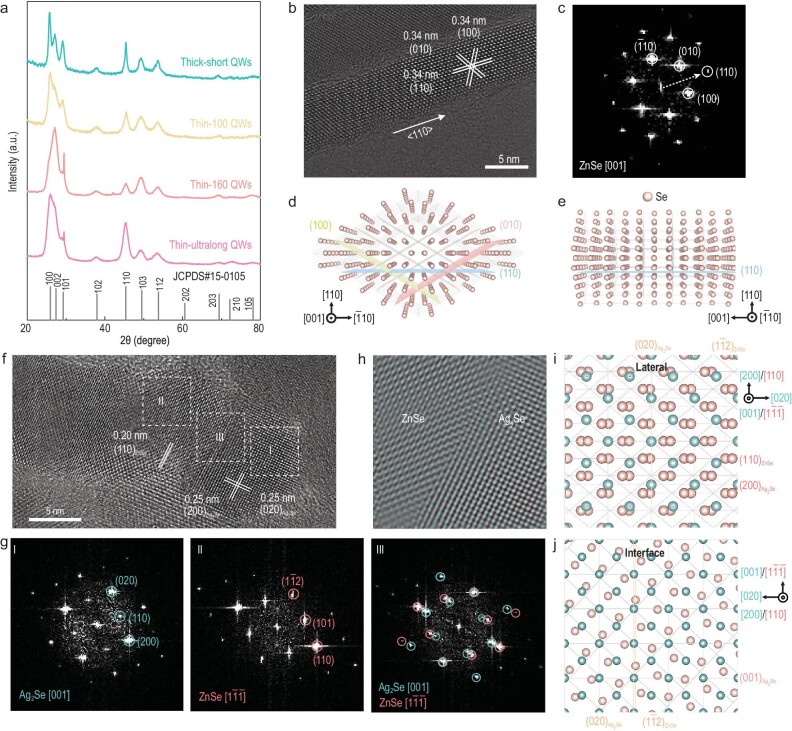
Crystal structure characterization of ZnSe QWs. (a) XRD patterns of ZnSe QWs with varied sizes, demonstrating their wurtzite crystal structure. (b) Representative HRTEM image of thin-100 ZnSe QW, revealing the high crystallinity without stacking faults. (c) FFT image from region in (b), highlighting the unique growth direction along the <110>_wz_ axis. (d) and (e) Anion sub-lattice models of ZnSe QWs viewed along the [001] (d) and [${\rm{\bar{1}}}$10] (e) zone axes, according well with the FFT image. (f) HRTEM image of the Ag_2_Se-tipped ZnSe QW. (g) FFT images from different regions in (f), as marked with dashed squares, which correspond to Ag_2_Se tip (I), ZnSe QW (II) and their heterointerfaces (III), respectively. The overlapped diffraction spots confirm the high-degree epitaxial growth between *bcc*–Ag_2_Se and *wz*–ZnSe, wherein the [001] zone axis of Ag_2_Se parallels well with the [1${\rm{\bar{1}\bar{1}}}$] axis of ZnSe. (h) Superimposed color-coded inversed FFT image from (g-III) using masked spots. (i) and (j) Overlapped anion sub-lattice models of Ag_2_Se (cyan) and ZnSe (pink) viewed in the lateral (i) and along the normal of the interface (j), highlighting their epitaxial relationship.

Detailed structural characterization then enabled us to better understand the growth mechanism of such high-quality QWs. As shown in the powder X-ray diffraction patterns (Fig. 4a), all ZnSe QWs possess the hexagonal wurtzite (WZ) structure (JCPDS Card, No. 15-0105). The representative high-resolution TEM (HRTEM) images further substantiate the high crystallinity of these ZnSe QWs without observable stacking faults (Fig. 4b and Supplementary Fig. 16). The typical hexagonal patterns comprising a set of {100} lattice planes in the HRTEM images accord well with the high intensity of (100) diffraction peaks in the X-ray diffraction (XRD) patterns. Corresponding fast Fourier transform (FFT) images (Fig. 4c) and oriented anion sub-lattice models (Fig. 4d and e) indicate that the ZnSe QWs epitaxially grow along the <110>_ __WZ_ crystallographic direction. This is surprising and not reported in prior wurtzite ZnSe nanowires, which usually adopted the <001>_ WZ_ polar axis [24,30,38].

To decipher the epitaxial relationship that leads to such a new growth direction, we collected the HRTEM image at the catalyst–QW interface (Fig. 4f). Combining FFT images from three different regions of heterostructures—the Ag_2_Se tip (Fig. 4g-I), ZnSe QWs (Fig. 4g-II) and the Ag_2_Se–ZnSe interface (Fig. 4g-III)—we confirmed the lattice-matched epitaxy between the body-centered cubic (*bcc*) Ag_2_Se catalyst and the hexagonal-phase ZnSe QW, which form an atomically sharp heterointerface. The observation of the cubic Ag_2_Se is reasonable taking into account the catalytic mechanism in solution–solid–solid growth, wherein the low-temperature monoclinic Ag_2_Se transforms into a cubic-phase superionic one above its phase-transition temperature (typically 110^o^C for bulk Ag_2_Se) [29]. Reversed FFT images further verify their epitaxial relationship (Fig. 4h and Supplementary Fig. 17). Corresponding anion sub-lattice models of *bcc*–Ag_2_Se and *wz*–ZnSe accord well with those FFT images, further substantiating the feasibility of such unique epitaxial growth (Fig. 4i and j). The high degree of epitaxy along the <110> axis instead of the <001> axis in wurtzite structures effectively eliminate the stacking faults ubiquitously observed in II–VI semiconductor nanowires. This, in turn, further circumvents mixed-phase induced inhomogeneous broadening of the optical absorption spectra. Overall, the high quality of ZnSe QWs with a unique growth direction leads to the ultranarrow absorption FWHM in the blue-light region.

### Solar-to-hydrogen production and underlying photophysics

Thanks to their environmental benignity and suitable bandgap for blue-light harvesting, the heavy-metal-free ZnSe has been of wide interest for solar-to-fuel conversion. Meanwhile, beyond the diameter-dependent band structures and optical absorption range, the quantum confinement effect in these ZnSe QWs can further enable fast interfacial charge transfer owing to the increased energies and densities of confined electron–hole pairs, which is benefitial for solar-to-fuel conversion. The conduction band minimum (CBM) of bulk ZnSe is energetically higher than the H_2_/H^+^ redox potential, suggesting that it could be a potential photocatalyst of high efficiency for solar-driven hydrogen production [40]. We thus used 3-mercaptopropionic acid (MPA), a typical amphiphilic molecule, to transfer these QWs from chloroform to water [41]. The resultant ZnSe QWs were well dispersed in water with superior environmental stability against oxidation (Supplementary Fig. 18); the phase-transfer process showed negligible effects on their optical absorption spectra (Supplementary Fig. 19). In the absence of any co-catalysts, these plain QWs exhibited superior activities in photocatalytic hydrogen evolution that exceeded 3 mmol h^–1^ g^–1^ (Supplementary Fig. 20 and Supplementary Table 5). In contrast, Ag_2_Se-tipped ZnSe QWs exhibited moderate photocatalytic performances (Supplementary Fig. 21), which may result from the type-I band alignment and the short-lived carriers in Ag_2_Se tips. The above results substantiate the utility of these quantum-confined, blue-light-active ZnSe QWs, underlining their potentials for specific blue-light-driven photochemical reactions.

**Figure 5.**
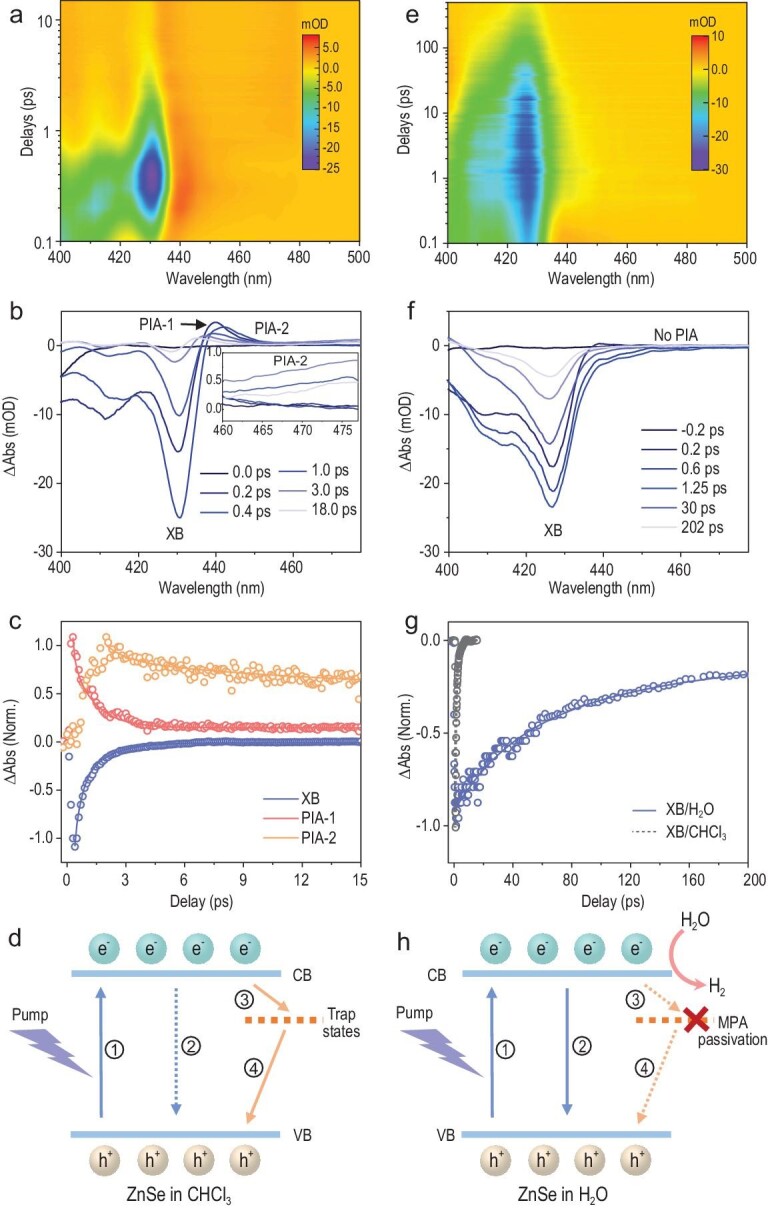
Transient absorption (TA) spectra and underlying photophysics of ZnSe QWs. (a) Pseudocolor plot of the TA spectra pumped at 375 nm for thin-100 ZnSe QWs in CHCl_3_. (b) TA spectra of thin-100 ZnSe QWs at indicated time delays, presenting distinct features of exciton bleach (XB) and photoinduced absorption (PIA-1 and PIA-2). Inset: the zoom-in view of the broad PIA-2 signal. (c) TA kinetics for three spectral features, monitored at 430 nm (XB), 440 nm (PIA-1) and 475 nm (PIA-2), respectively. (d) Schematic energy levels and exciton quenching pathways in ZnSe QWs before MPA passivation. (e) Pseudocolor plot of the TA spectra pumped at 375 nm for MPA-capped thin-100 ZnSe QWs in H_2_O after ligand exchange. (f) TA spectra of MPA-capped thin-100 ZnSe QWs at indicated time delays, implying a lack of PIA signals. (g) TA kinetics of XB signals for ZnSe QWs in CHCl_3_ and H_2_O, indicating order-of-magnitude enhancement of the 1S_e_ lifetime after MPA passivation. (h) Schematic energy levels and exciton quenching pathways in ZnSe QWs after MPA passivation.

We then sought to investigate the carrier dynamics of ZnSe QWs using transient absorption (TA) spectroscopy. Figure 5a and b present the typical TA spectra of ultrathin ZnSe QWs as pumped at 375 nm, which display multiple bleach features at different exciton transition energies—a consequence consistent with their steady-state absorption spectra. Owing to the higher degeneracy of the valence band and larger effective mass of holes, the exciton bleach (XB) at 430 nm is dominantly attributed to the state filling of the 1S_e_ excited state [42–44]. Strikingly, we found that the XB decays extremely fast with a lifetime of only sub-2 ps (Fig. 5c), contradicting with their high photocatalytic performances.

In addition to the XB signals, there are two distinguished photoinduced absorption (PIA) signals: one at the red side of XB with derivative-like features (PIA-1), the other with a broad band beyond 460 nm (PIA-2). The PIA-1 feature at ∼440 nm typically arises from hot electrons (at the early rise time) and the biexciton effect in the ZnSe QWs, as confirmed by the consistent formation and decay dynamics of XB and PIA-1 signals [45]. The broad PIA-2 signal usually arises from carriers trapped by mid-gap surface states [46]. In conventional Cd-containing II–VI semiconductors, this PIA-2 signal was attributed to trapped holes, which should appear concomitantly with the XB signal generation due to the exciton dissociation and hole transfer to hole-trapping states. Thus, the hole-trapping process is expected to generate long-lived electrons in the 1S_e_ states [44]. However, in our case, the XB decays rather fast with PIA-2 dynamics out of sync, which is quite different from the hole-trapping scenario. Importantly, the PIA-2 forms instantly after the decay of XB signals with the same timescale, suggesting that PIA-2 arises, instead, from trapped electrons by surface states (Fig. 5c). The electron-trapping process also interprets well the ultrafast decay of the XB signal (Fig. 5d). The existence of electron-trapping states instead of hole-trapping ones is rational, considering the fact that the CBM and valence-band maximum in bulk ZnSe are close to the vacuum level, such that electron-trapping surface states can easily locate within the band gap while hole-trapping ones tend to locate within the valence band [40]. Further TA characterization on other QWs shows that the thin-100 and thin-160 QWs present well-defined exciton bleach features due to their strong quantum confinement effect (Supplementary Fig. 22). The positions of XB signals in these QWs accord well with their optical absorption spectra in Fig. 3. The TA kinetics of XB signals for ZnSe QWs of other sizes present short-lived XB with apparent PIA-2 signals as well, implying similar electron-trapping processes in these QWs.

The contradictory results between photocatalytic performances and carrier dynamics in chloroform motivated us to further examine the TA spectra in water. As shown in Fig. 5e–g and Supplementary Fig. 23, all ZnSe QWs in water demonstrate a several-orders-of-magnitude longer lifetime for the 1S_e_ excited state with no observable PIA signals. The diminishing surface electron traps result from the excellent electron-donating ability of the thiol group in MPA ligands [47]. Consequently, the long-lived electrons in the CBM give rise to high photocatalytic performances (Fig. 5h). These results suggest the general effect of MPA passivation on eliminating surface electron traps in ZnSe QWs. This translates into a set of surface-engineerable ZnSe QWs that would offer extensive opportunities for blue-light-related photoelectric and photochemical conversion.

## CONCLUSION

In summary, we report a two-step catalyzed growth strategy that enables simultaneous, on-demand and high-precision control over the radial and axial size of ZnSe QWs, which feature well-defined exciton transitions in the blue-light region. This work bridges the gap between prior magic-sized QWs and bulk-like nanowires; the former show limited size tunability and inaccessible blue-light absorption, while the latter are beyond the quantum-confined regime. The high quality of such ZnSe QWs yields ultra-narrow exciton transition linewidth and remarkably high performances in solar-to-fuel conversion. These findings highlight such heavy-metal-free QWs as exciting platforms for blue-light-active applications spanning from polarized light-emitting devices to solar energy conversion.

## METHODS

### Size-controlled synthesis of Ag_2_Se-tipped ZnSe QWs

First, a Se–OAm precursor was prepared by heating the mixture of 1 mmol selenium powder with 10 mL oleylamine (C_se _= 0.1 mM) at 250^o^C for 30 min, forming a clear solution. Then, Ag_2_Se nanocrystals were synthesized via direct reaction of AgNO_3_ and Se–OAm precursor in oleylamine according to the literature-reported method [24]; the detailed synthetic process is described in the Supplementary Notes. A certain amount of Ag_2_Se reaction solution was precipitated by acetone and collected by centrifugation, and then immediately dispersed in 1 mL of oleylamine as the stock solution of catalytic seeds. Typically, for the thick-short QWs (8.5 ± 1.1 × 45 ± 10 nm), the volume of Ag_2_Se reaction solution required to be purified is 3 mL. First, 1 mL of Ag_2_Se/oleylamine stock solution was mixed with 9 mL of Se–OAm precursor. Next, the mixture was heated to 120^o^C and degassed for 20 min to remove water and oxygen. After that, the reaction solution was further heated to 210^o^C in 10 min under the nitrogen atmosphere and kept at 210^o^C for 8 min, by which the Ag_2_Se seeds ripened to larger sizes. Then, 40 mg of powdered zinc acetate was quickly added to the reaction solution by opening the glass stopper temporally and the reaction was kept for 3 min for QW growth. The products were collected and washed with hexane and ethanol twice for further use.

### Photocatalytic hydrogen evolution experiment

The hydrophobic ZnSe QWs were first transferred from hydrophobic to hydrophilic media using the ligand exchange method [41]. Photocatalytic reactions were carried out in the Labsolar 6A online photocatalytic system (Beijing Perfectlight Technology Co., Ltd). For each reaction, the ZnSe photocatalyst (10 mg) was dispersed in an aqueous solution (100 mL) containing Na_2_S (0.25 M) and Na_2_SO_3_ (0.35 M) as hole scavengers. Then, the reactor containing the reaction solution was evacuated several times and filled with nitrogen before full-spectrum irradiation under a 300-W Xe lamp. The reaction solution was maintained at 25^o^C by the circulating cooling water. The evolved hydrogen was analysed online using a gas chromatograph (Agilent 7890B) equipped with a thermal conductivity detector.

### TA spectroscopy measurements

The 1030-nm fundamental (5 kHz) was generated from a Yb : KGW regenerative amplifier (Pharos, Light Conversion). A portion of this beam was sent through an optical parametric amplifier (Orpheus, Light Conversion) to produce a photoexcitation pulse at 375 nm (pulse duration ∼250 fs). Both the photoexcitation and fundamental were sent into an optical bench (Helios, Ultrafast). After passing through a delay stage, the fundamental was focused into a sapphire crystal in order to generate the probe as a white-light continuum. The frequency of the pump pulse was reduced to 2.5 kHz using a chopper and the power of the pump pulse was kept sufficiently low in case of any sample damage. The two beams were then focused onto the sample, which was filled into a 1-mm cuvette. The differential absorption of the probe was then detected using a charge-coupled device (Helios, Ultrafast).

## Supplementary Material

nwac025_Supplemental_File
