# Supplementary material for: On-demand synthesis of high-quality, blue-light-active ZnSe colloidal quantum wires
Source: Natl Sci Rev. 2022 Feb 26;9(10):nwac025. doi: 10.1093/nsr/nwac025 (PMC9671665; doi:10.1093/nsr/nwac025)
Supplement: nwac025_Supplemental_File [file nwac025_supplemental_file.docx]

**Supplementary Information**

**On demand defining high-quality, blue-light-active ZnSe colloidal quantum wires**

Yi Li^1,🟉^, Chong Zhang^1,🟉^, Jie Tian^2^, Liang Wu^1^, Guo-Qiang Liu^1^, Hui-Hui Li^1^, Yu-Zhuo Zhang^1^, Zhen-Chao Shao^1^, Zhen He^1^, and Shu-Hong Yu^1,*^

^1^ Division of Nanomaterials & Chemistry, Hefei National Laboratory for Physical Sciences at the Microscale, CAS Center for Excellence in Nano science, Hefei Science Center of CAS, Department of Chemistry, University of Science and Technology of China, Hefei 230026, China

^2^ Engineering and Materials Science Experiment Center University of Science and Technology of China Hefei, Anhui 230026, China

^🟉^ Y.L. and C.Z. contributed equally to this work.

^*^ To whom correspondence should be addressed. Email: shyu@ustc.edu.cn.

**Supplementary Notes**

**Materials and Methods**

Silver nitrate (AgNO_3_, 99.8%), sodium hydroxide (NaOH, 96.0%), sodium sulfite (Na_2_SO_3_), zinc nitrate (Zn(NO_3_)_2_∙6H_2_O, 98%), zinc stearate (CP), hexane, methanol, ethanol, acetone, toluene, and chloroform (CHCl_3_) were purchased from the Sinopharm Chemical Reagent. Selenium power (99.9%), oleylamine (OAm, 80-90%), zinc acetate (Zn(Ac)_2_, 99.99%), selenium dioxide (SeO_2_, 99.9%), tributylphosphine (TBP, 98%), and sodium sulfide nonahydrate (Na_2_S∙9H_2_O, 98.0%) were purchased from Aladdin Chemicals. 3-mercaptopropionic acid (MPA) was purchased from Alfa Chemicals. Zinc acetylacetonate (99%) was purchased from J&K Chemical Ltd. All chemicals were used as received without any further purification.

**Synthesis of catalyst Ag_2_Se nanocrystals**

The Ag_2_Se nanocrystals were synthesized via direct reaction of AgNO_3_ and Se-OAm precursor in oleylamine. Typically, to synthesize Ag_2_Se nanoparticles with averaging diameter of 5.4 nm, oleylamine (8 mL) in the three-necked flask was degassed at 120 ^o^C for 30 min to remove water and oxygen. Once AgNO_3_ (34 mg) was added into the solution by opening the glass stopper temporally, fresh Se-OAm precursor (2 mL) was then immediately (within 10 sec) injected into the solution. The color of the reaction solution changed from pale yellow to jet black, indicating the formation of Ag_2_Se nanoparticles. The solution was further kept at 120 ^o^C for 90 min under nitrogen atmosphere to obtain nanocrystals of desired sizes. Ag_2_Se nanoparticles of different sizes can be obtained similarly by varying the reaction temperature and growth time.

**Synthesis of ZnSe QWs**

For the thin-100 QWs (4.5±0.3 nm×95±11 nm), the reaction was the same as that for thick-short QWs expect that the Ag_2_Se stock solution was added until 9 mL Se-OAm was heated to 210 ^o^C, after which 40 mg Zn(Ac)_2_ was immediately added. The reaction was kept for certain time to obtain QWs of desired length.

For the thin-160 QWs (4.9±0.4 nm×159±23 nm), the reaction was similar to the case of thin-100 QWs, except that 1 mL of Ag_2_Se reaction solution and 60 mg Zn(Ac)_2_ were used instead.

For the thin-ultralong QWs (5.9±0.7 nm in diameter), SeO_2_ was used instead of Se-OAm as the Se precursor. The reaction was similar to the case of thin-100 QWs, except that 1.5 mL Ag_2_Se reaction solution, 120 mg Zn(Ac)_2_, and 44.4 mg SeO_2_ were added successively into the reaction solution in a one-off manner when 9 mL OAm was heated to 210 ^o^C.

Plain ZnSe QWs of different sizes were obtained by removing the Ag_2_Se tips of above QWs using alkylphosphine. Typically, the synthesized Ag_2_Se-ZnSe QWs were well dispersed in 20 mL toluene, and then mixed with 2 mL TBP and 10 mL methanol. The solution gradually turned yellow after magnetic stirring for several hours. The final QWs were collected by centrifugation and washed twice with hexane and ethanol twice for further use.

**Characterization**

The powder X-ray diffraction (PXRD) measurements were conducted on a X-ray diffractometer (Philips) using graphite-monochromatized Cu Kα radiation. For morphological characterization, transmission electron microscopy (TEM) images and high-resolution transmission electron microscopy (HRTEM) images were collected on Hitachi H-7700 (Japan) and JEOL-2100F (Japan) with an acceleration voltage of 100 kV and 200 kV, respectively. High-angle annular dark field scanning transmission electron microscopy (HAADF-STEM) images and EDS spectra were collected on Talos F200X (FEI Inc.) with operation voltage of 200 kV. UV-vis absorption spectra of ZnSe QWs were collected on UV-2600 (Shimadzu Corporation, Japan). To quantify the element contents in each QWs, at least three batches of inductively coupled plasma atomic emission spectroscopy (ICP-AES) tests were carried out on Optima 7300 DV to ensure the reliability. X-ray photoelectron spectra (XPS) were recorded on a Thermo ESCALab 250Xi spectrometer with an excitation source of monochromatized Al Kα radiation (1486.6 eV) and a pass energy of 30 eV, where the binding energies were calibrated with the C1s peak of contaminant carbon at 284.80 eV.

**Supplementary Figures**


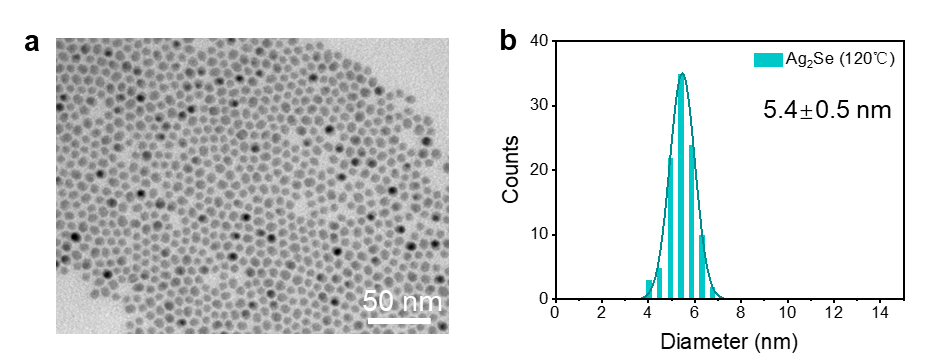


**Supplementary Fig. 1.** Morphological characterization of Ag_2_Se catalyst seeds. (a) TEM image and (b) corresponding statistical size distribution of Ag_2_Se nanoparticles synthesized at 120^o^C for 90 min.


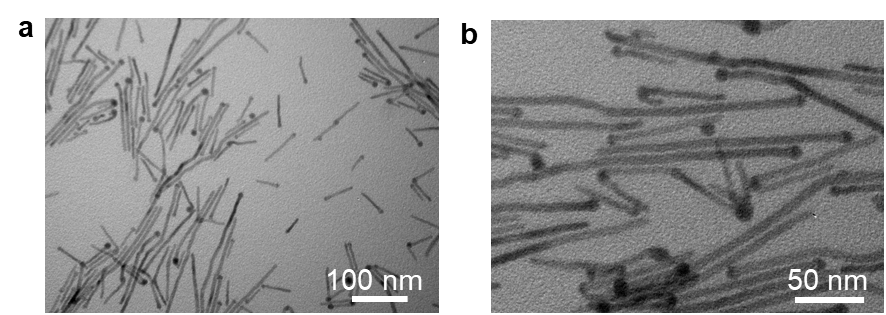


**Supplementary Fig. 2.** Ag_2_Se-ZnSe QWs using colloidal Ag_2_Se stock solution without purification. (a) Low-magnification TEM image. (b) High-magnification TEM image. In this case, uncontrolled ripening of catalysts and inhomogeneous growth of QWs occurred.


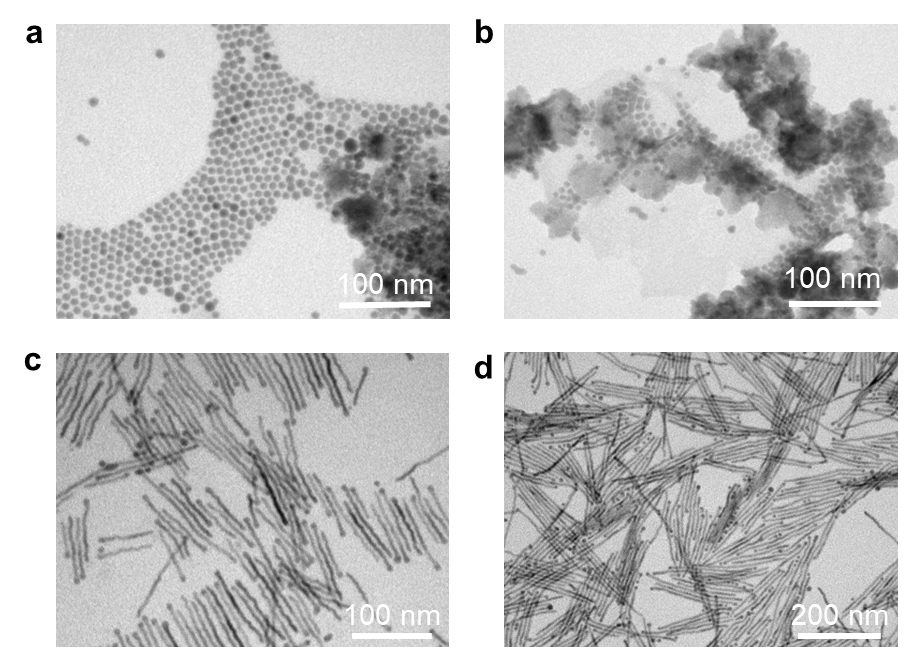


**Supplementary Fig. 3.** TEM images of products for ZnSe growth using different amount of Se-OAm precursor. (a) 1 mL Se-OAm. (b) 2 mL Se-OAm. (c) 9 mL Se-OAm. (d) 14 mL Se-OAm. With low amount of Se precursor (sub-stoichiometric Se/Zn ratio), the catalyzed growth is inhibited; while using Se precursor more than 9 mL, the quality of as-synthesized ZnSe QWs shows no difference from that with 9 mL.


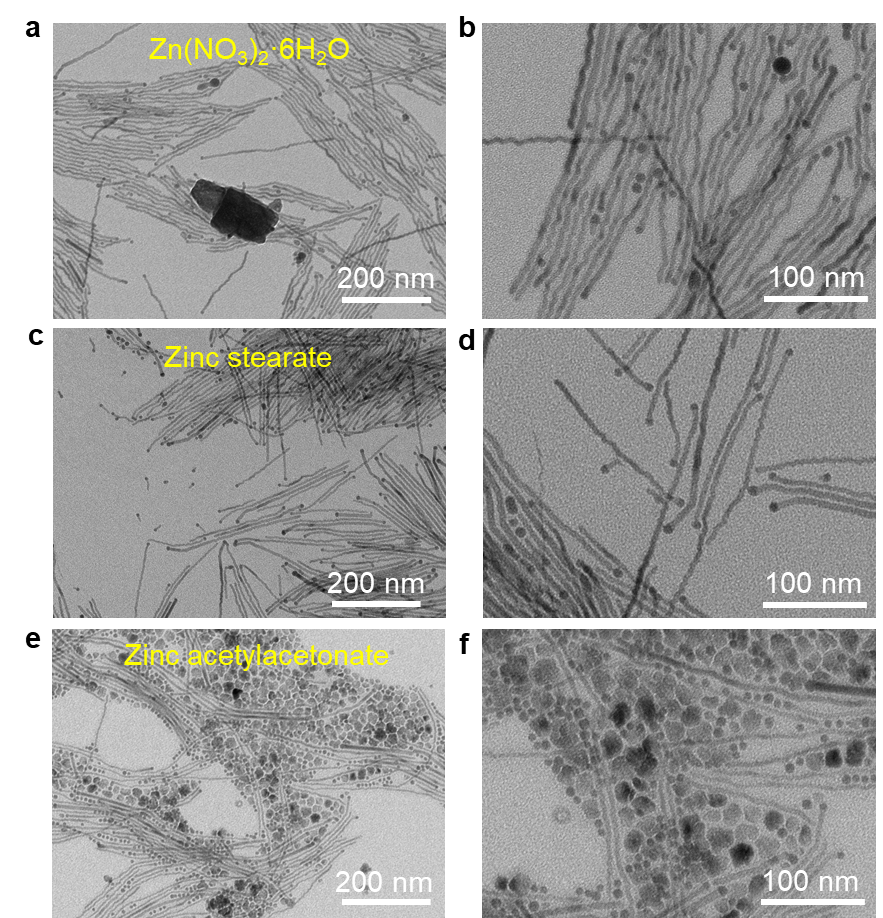


**Supplementary Fig. 4.** Low- and high-magnification TEM images of products for ZnSe nanowires using different kinds of Zn precursors. (a,b) Zn(NO_3_)_2_·6H_2_O. (c,d) Zinc stearate. (e,f) Zinc acetylacetonate. ZnSe nanowires obtained from Zn(NO_3_)_2_·6H_2_O and zinc stearate are of poor quality and non-uniformity in length; while zinc acetylacetonate results in by-products of nanoparticles. The synthetic procedure is the same as that for thin-160 QWs except Zn precursors.


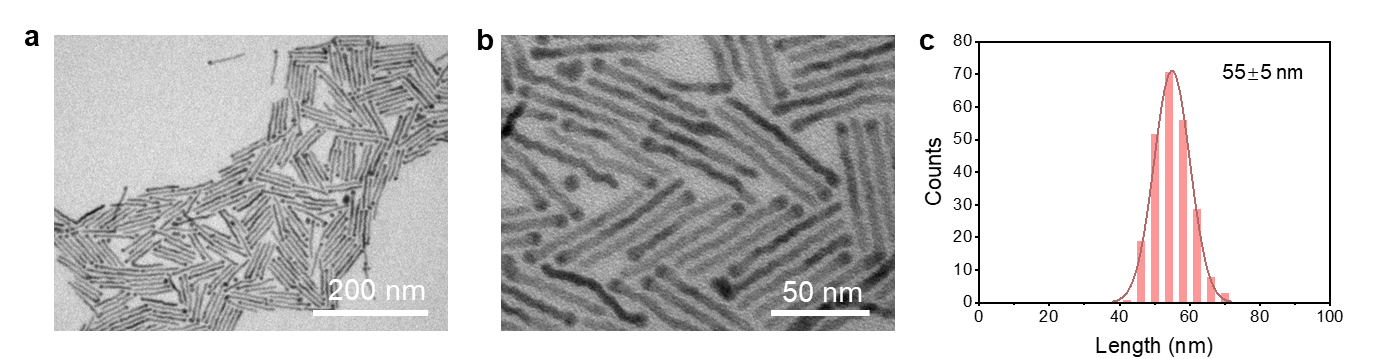


**Supplementary Fig. 5.** Morphological characterization of thin-short Ag_2_Se-ZnSe QWs with shorter growth time. (a) Low- and (b) high-magnification TEM images of thin-short Ag_2_Se-ZnSe QWs. (c) Corresponding statistical diameter distributions. The synthetic procedure is the same as that for thin-100 QWs except with shorter growth time of only 10 seconds.


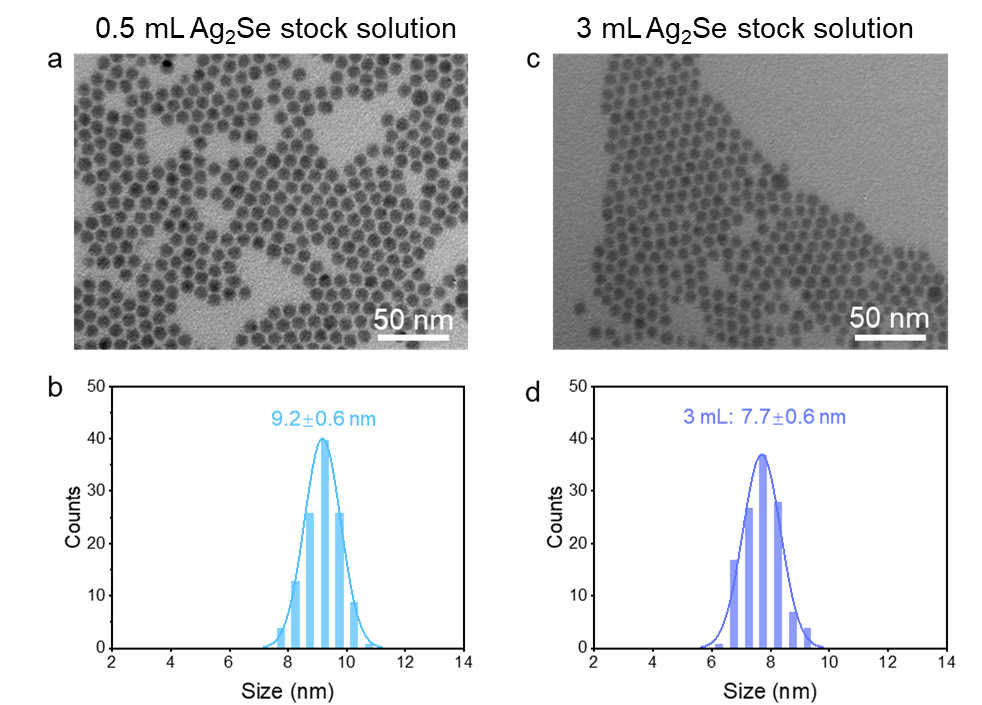


**Supplementary Fig. 6.** Size modulations of Ag_2_Se catalysts by changing the amount of seeds injected. (a,b) TEM image (a) and corresponding diameter distributions (b) of 0.5 mL Ag_2_Se seeds stock solution after ripening at 210 ^o^C for 3 min. (c,d) TEM image (c) and corresponding diameter distributions (d) of 3 mL Ag_2_Se seeds stock solution after ripening at 210 ^o^C for 3 min.


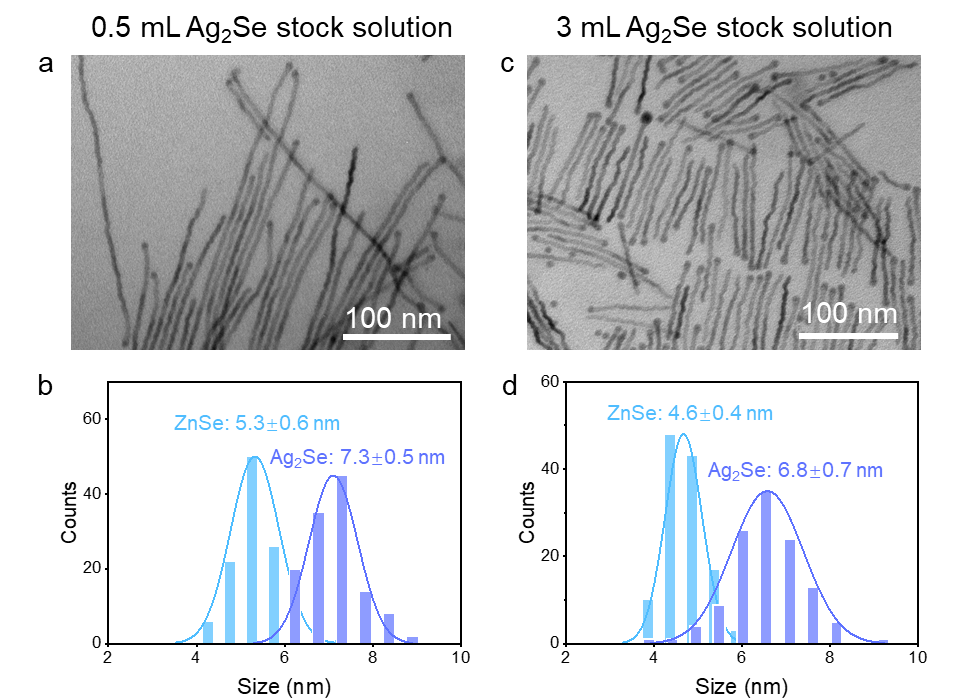


**Supplementary Fig. 7.** Size modulations of both Ag_2_Se tips and ZnSe QWs by changing the amount of seeds injected. (a,b) TEM image (a) and corresponding diameter distributions (b) of the resulting ZnSe QWs after injecting 0.5 mL Ag_2_Se seeds stock solution without the initial period of ripening. (c,d) TEM image (c) and corresponding diameter distributions (d) of the resulting ZnSe QWs after injecting 3 mL Ag_2_Se seeds stock solution without the initial period of ripening.


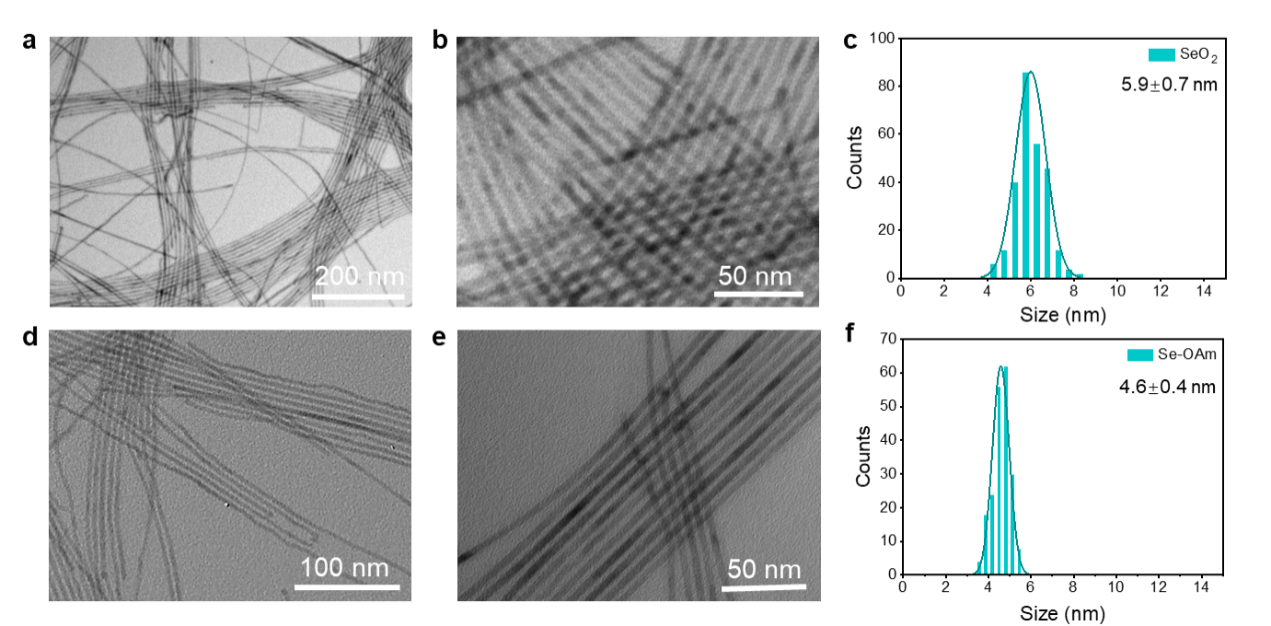


**Supplementary Fig. 8.** Morphological characterization of thin-ultralong Ag_2_Se-ZnSe QWs using different Se precursors. (a) Low- and (b) high-magnification TEM images of thin-ultralong Ag_2_Se-ZnSe QWs using SeO_2_ as the Se precursor. (c) Corresponding statistical diameter distributions of thin-ultralong Ag_2_Se-ZnSe QWs in (a). (d) Low- and (e) high-magnification TEM images of thin-ultralong Ag_2_Se-ZnSe QWs using using Se-OAm as the Se precursor. (f) Corresponding statistical diameter distributions of thin-ultralong Ag_2_Se-ZnSe QWs in (d).


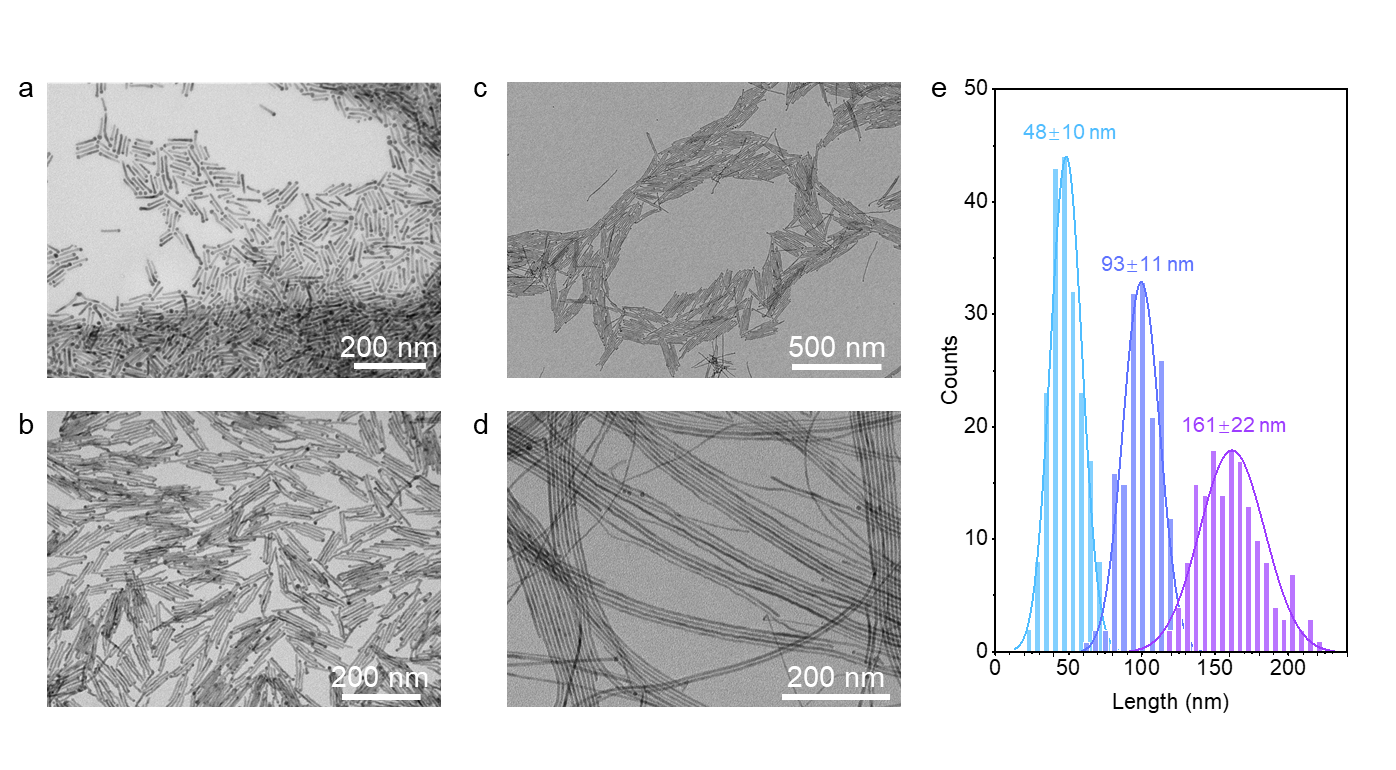


**Supplementary Fig. 9.** Low-magnification TEM images of different ZnSe QWs. (a) Thick-short QWs. (b) Thin-100 QWs. (c) Thin-160 QWs. (d) Thin-ultralong QWs. (e) Statistical length distributions of different ZnSe QWs, showing the high uniformity and reproducibility.


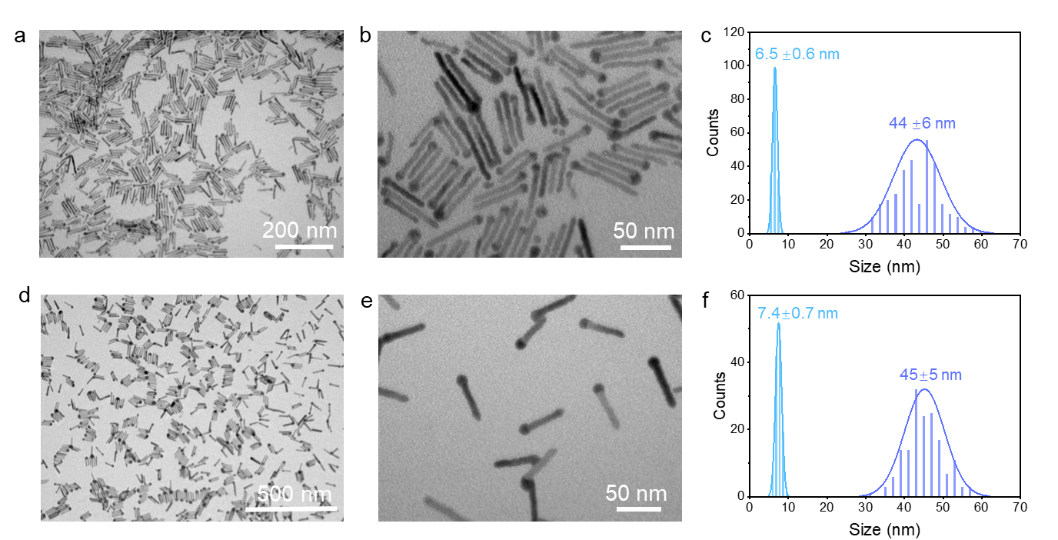


**Supplementary Fig. 10.** Two additional diameter modulations of ZnSe QWs by controlling the ripening time of Ag_2_Se catalysts. (a-c) Low-magnification (a) and enlarged (b) TEM images of 6.5-nm thick ZnSe QWs and their corresponding diameter and length distributions (c). The synthetic procedure is the same as that for thin-100 QWs except that keeping the Ag_2_Se seeds ripening for 4 min at 210 ^o^C, after which 40 mg Zn(Ac)_2_ was immediately added. (d-f) Low-magnification (d) and enlarged (e) TEM images of 7.4-nm thick ZnSe QWs and their corresponding diameter and length distributions (f). The synthetic procedure is the same as that for thick-short QWs except that the ripening time was reduced from 8 min to 4 min.


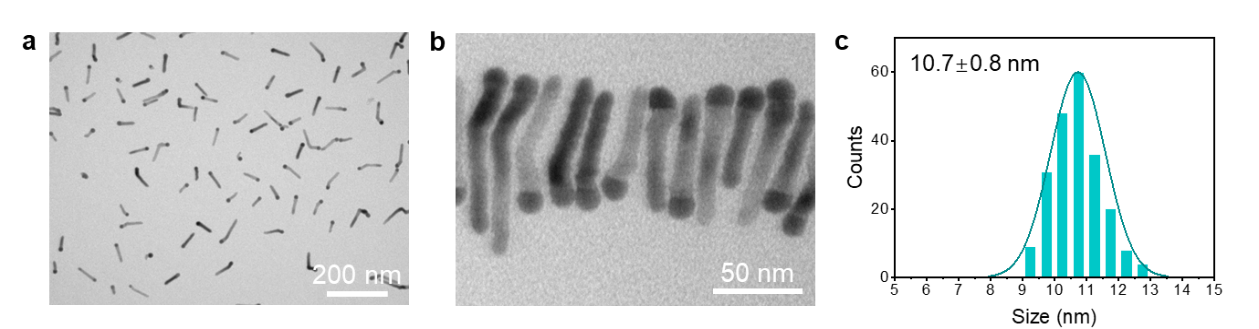


**Supplementary Fig. 11.** Morphological characterization of bulk-like thick ZnSe QWs using Ag_2_Se seeds of larger size. (a) Low- and (b) high-magnification TEM images of thick Ag_2_Se-ZnSe QWs using Ag_2_Se seeds synthesized at 160 ^o^C. (c) Corresponding statistical diameter distributions of thick Ag_2_Se-ZnSe QWs in (a). The synthetic procedure is the same as that for thick-short QWs except the size of Ag_2_Se seeds.


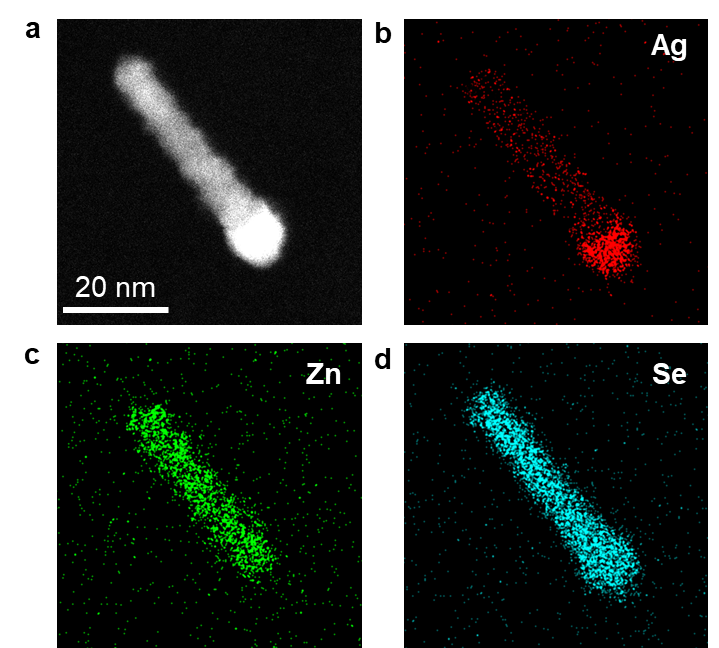


**Supplementary Fig. 12.** EDS elemental mapping images of thick-short Ag_2_Se-ZnSe nanowire.


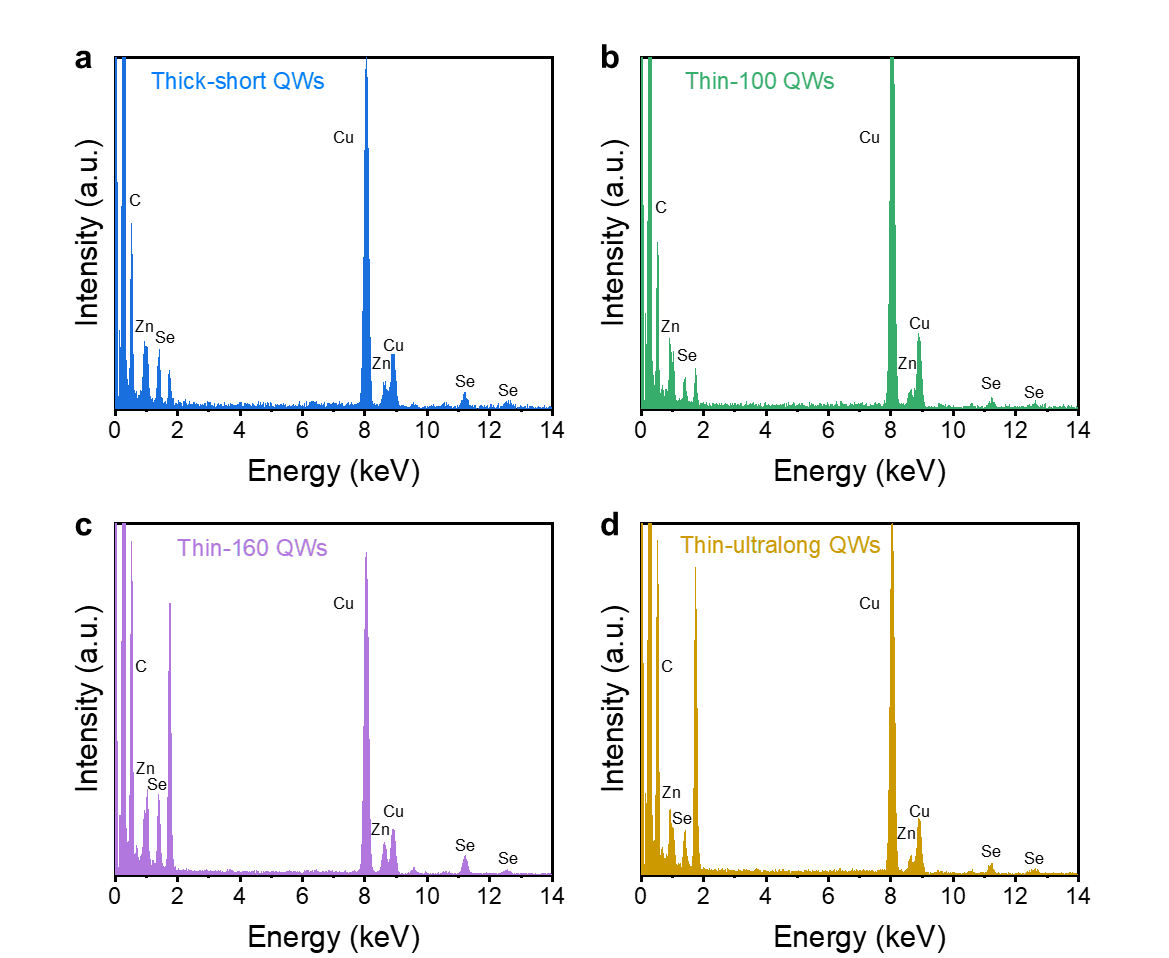


**Supplementary Fig. 13.** EDS spectra of ZnSe QWs. (a) Thick-short, (b) thin-100, (c) thin-160, and (d) thin-ultralong ZnSe QWs. The C and Cu signals come from the copper grid.


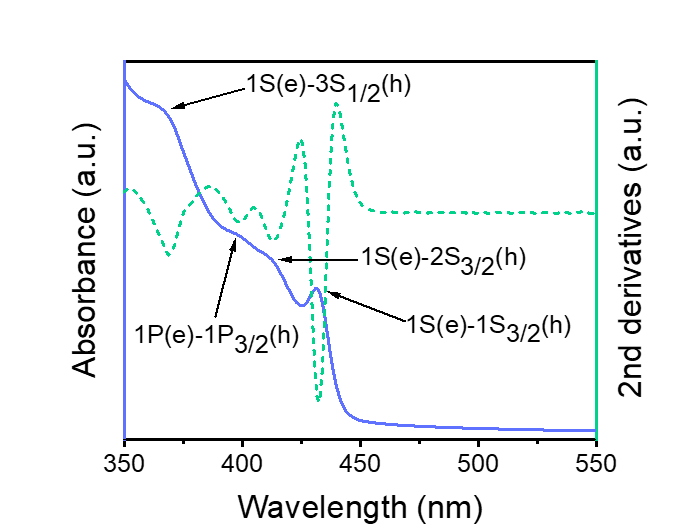


**Supplementary Fig. 14.** Assignment of each excitonic transition feature of the optical absorption spectrum (blue solid line) of the thin-100 ZnSe QWs in combination with its second derivatives (green dash line)[1].


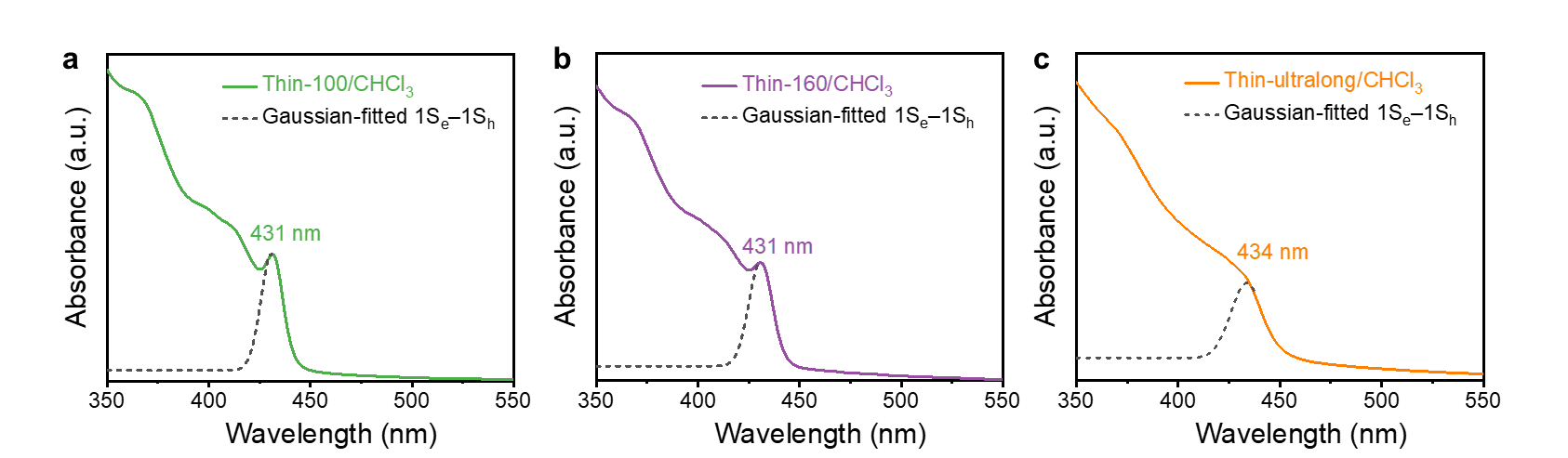


**Supplementary Fig. 15.** UV-vis absorption spectra (solid line) and Gaussian-fitted 1S_e_-1S_h_ exciton transitions (dash line) of ZnSe QWs with different sizes. (a) Thin-100 QWs. (b) Thin-160 QWs.


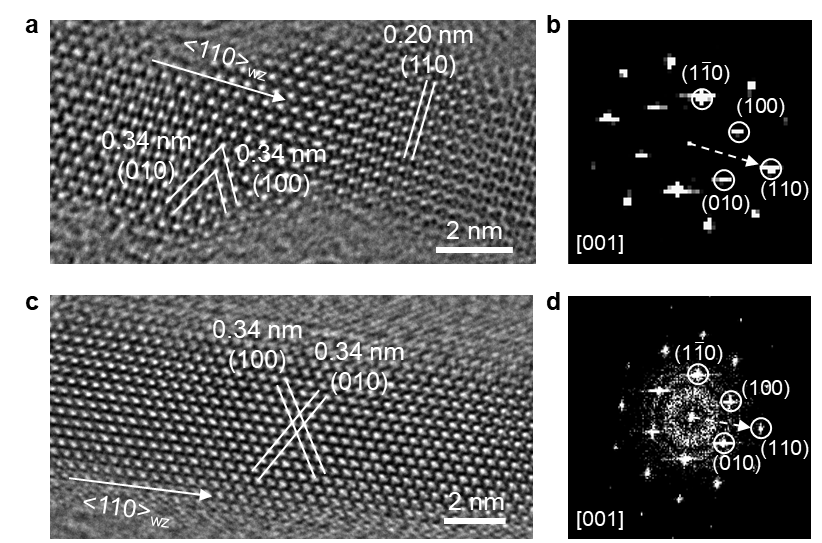


**Supplementary Fig. 16.** Crystal structure characterization of ZnSe QWs. (a) HRTEM image and (b) corresponding FFT image of thin-160 ZnSe QWs. (c) HRTEM image and (d) corresponding FFT image of thin-ultralong ZnSe QWs. All these results confirm that ZnSe QWs presented here adopt an unique epitaxial growth direction along the [110]_WZ_ axis.


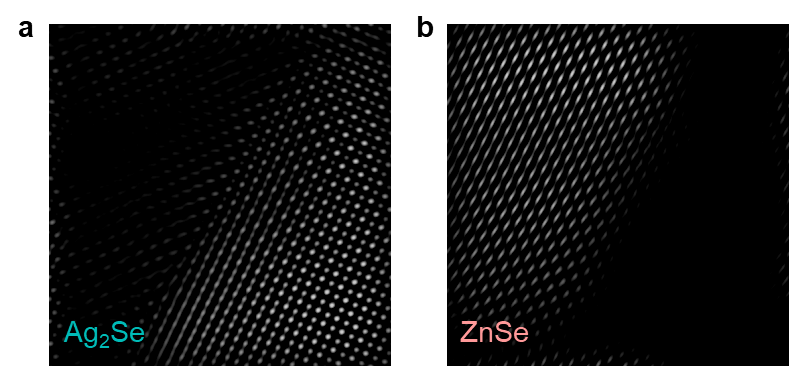


**Supplementary Fig. 17.** Individual inverse fast Fourier transformed images (FFTs) with masked spots on the HRTEM image of Ag_2_Se-ZnSe QWs in Figure 4f. (a) Ag_2_Se domain. (b) ZnSe domain.


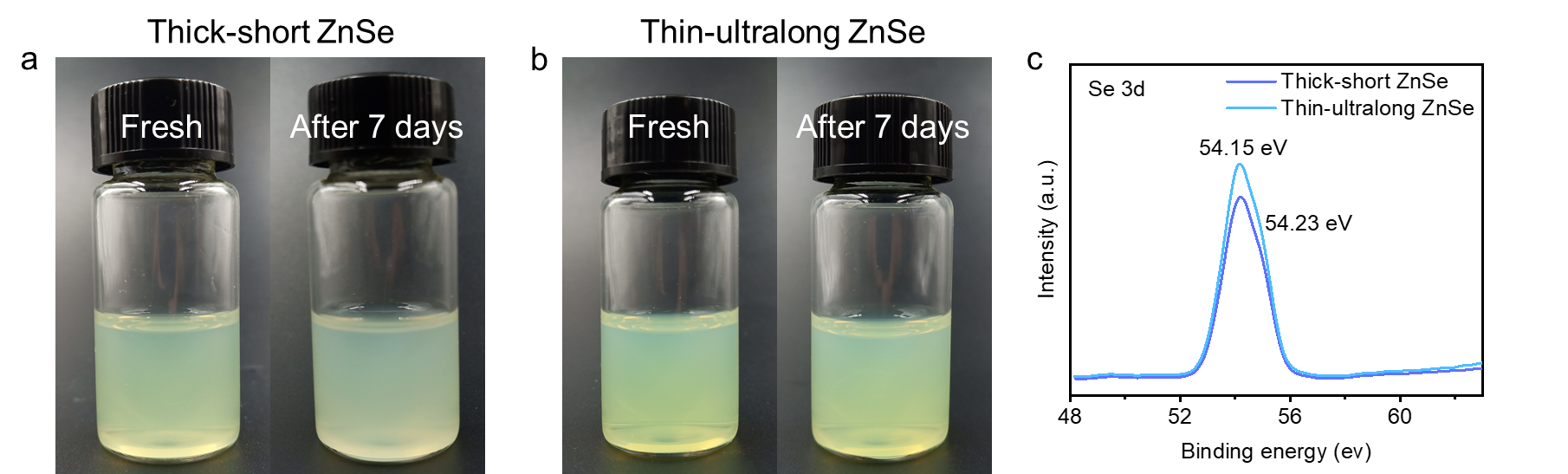


**Supplementary Fig. 18.** The environmental stability of MPA-passivated ZnSe QWs dispersed in aqueous solution. (a) Optical photos of freshly prepared thick-short ZnSe nanowires and those stored for 7 days. (b) Optical photos of freshly prepared thin-ultralong ZnSe nanowires and those stored for 7 days. (c) XPS spectra of Se 3d in ZnSe nanowires stored for 7 days.


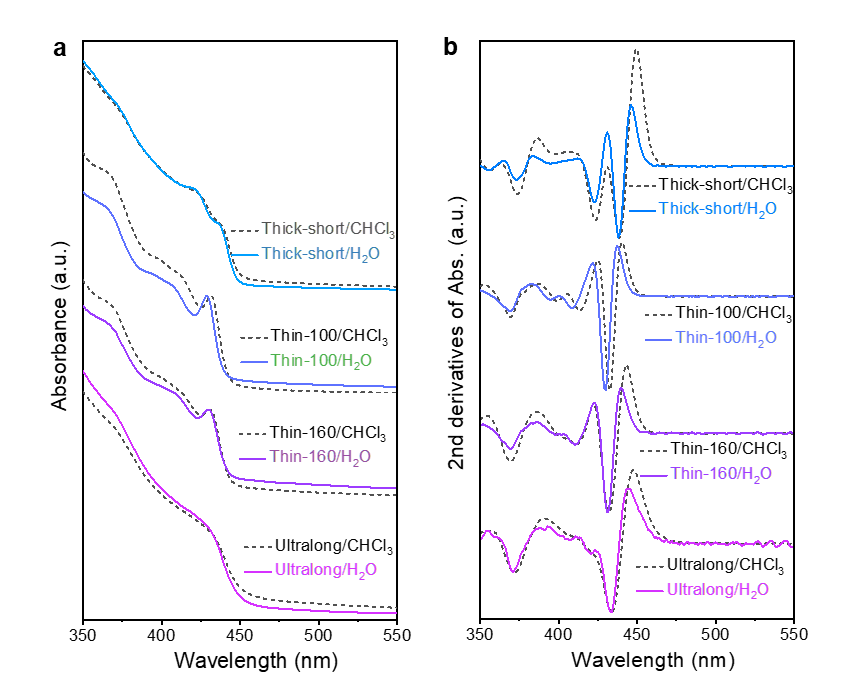


**Supplementary Fig. 19.** UV-Vis absorption spectra of ZnSe NWs. (a) UV-Vis absorption spectra of ZnSe NWs with different sizes, dispersed in deionized water (solid line) and CHCl_3_ (dash line). (b) Corresponding second derivatives of absorption spectra.


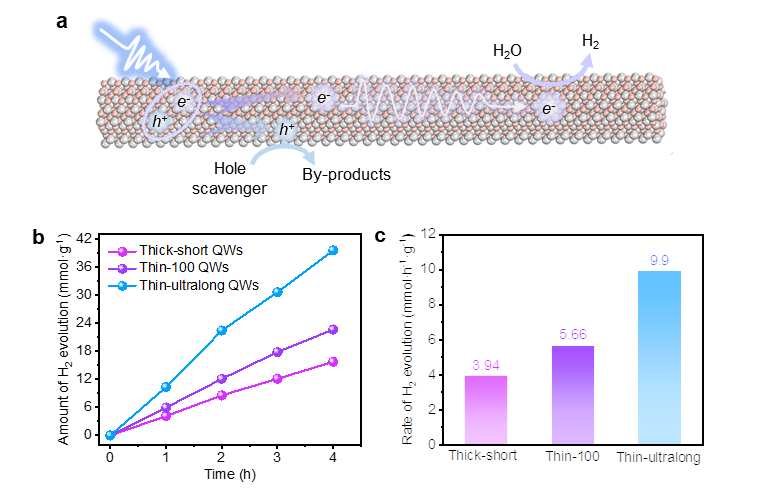


**Supplementary Fig. 20.** Photocatalytic hydrogen production performances of ZnSe QWs. (a) Schematic of photocatalytic H_2_ production from water using ZnSe QWs, which feature blue-light-active optical absorption, large absorption cross section, strong excitonic effect, efficient charge separation, and long-distance carrier transport. (b) Time-dependent H_2_ productions for different QWs. (c) Corresponding photocatalytic H_2_ production rates.


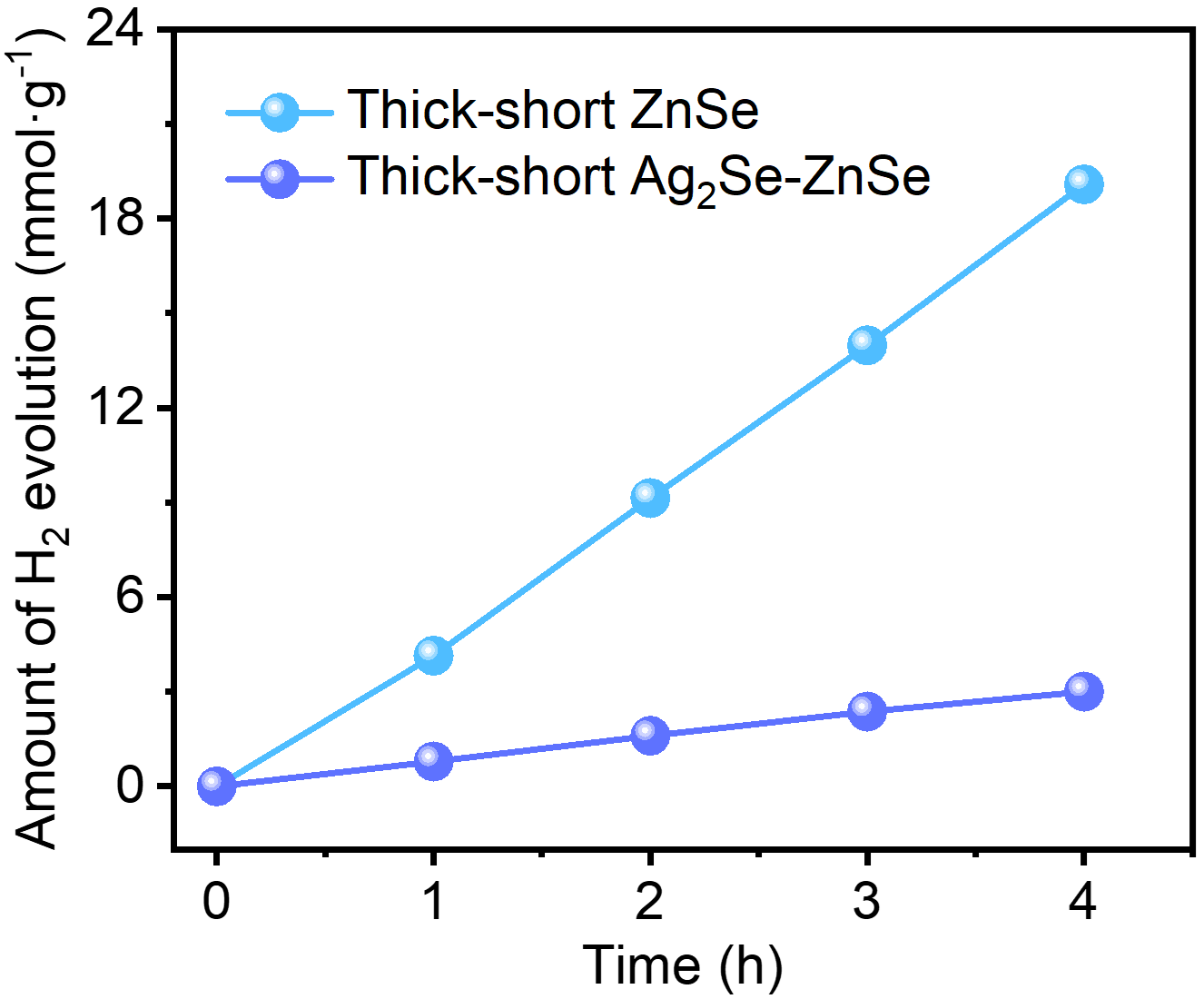


**Supplementary Fig. 21.** Photocatalytic performances of Ag_2_Se-tipped and plain ZnSe QWs.

**
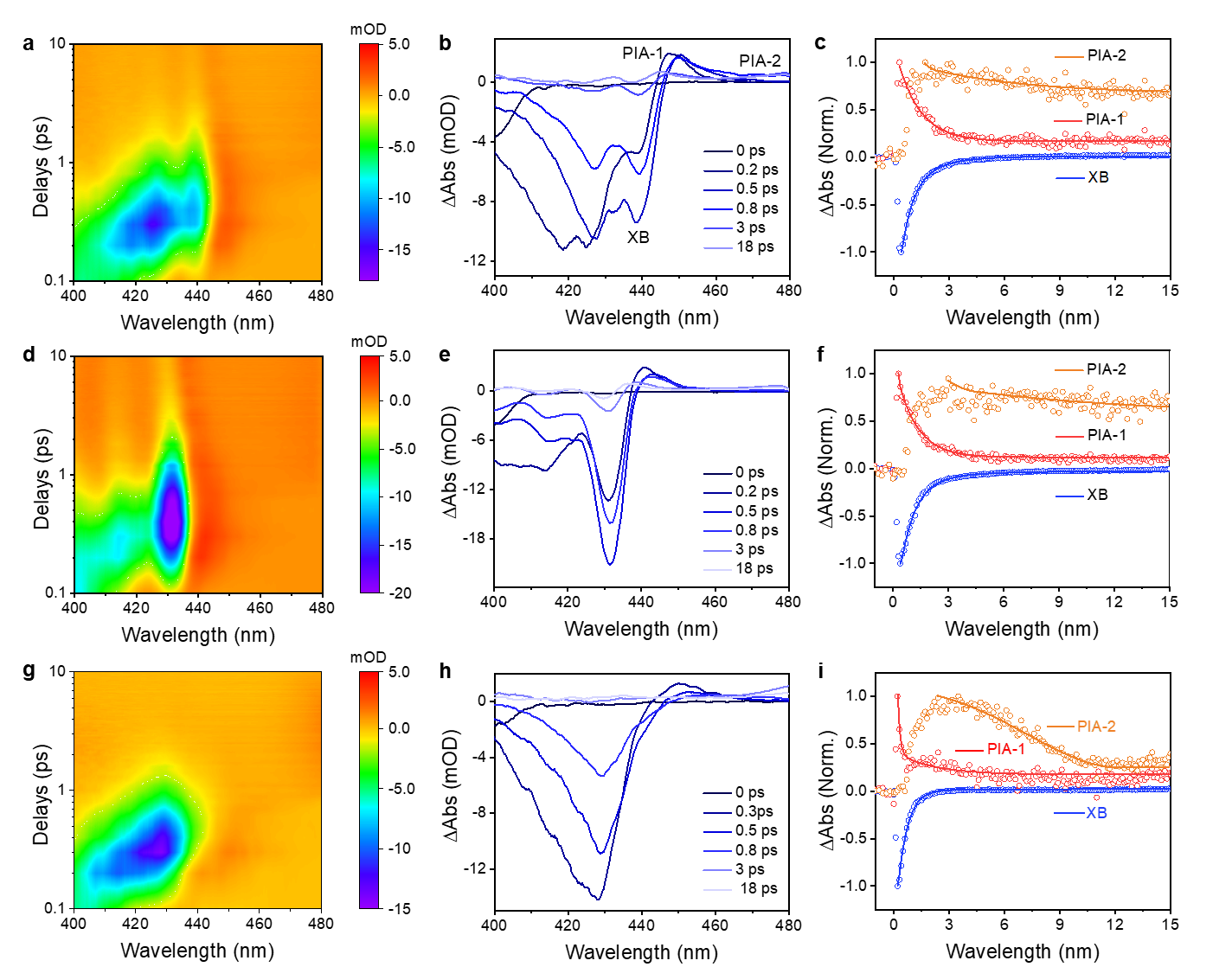
**

**Supplementary Fig. 22.** Transient absorption (TA) spectra of ZnSe QWs in CHCl_3_. Pseudocolour plots of the TA spectra pumped at 375 nm for (a) thick-short, (d) thin-160 and (g) thin-ultralongZnSe QWs, respectively. TA spectra of (b) thick-short, (e) thin-160, and (h) thin-ultralong ZnSe QWs at indicated time delays, presenting distinct features of exciton bleach (XB) and photoinduced absorption (PIA-1 and PIA-2). TA kinetics for three spectral features in (c) thick-short, (f) thin-160, and (i) thin-ultralong ZnSe QWs. The consistent results in all samples substantiate the general existence of surface electron traps that lead to rapid decay of XB signals.

**
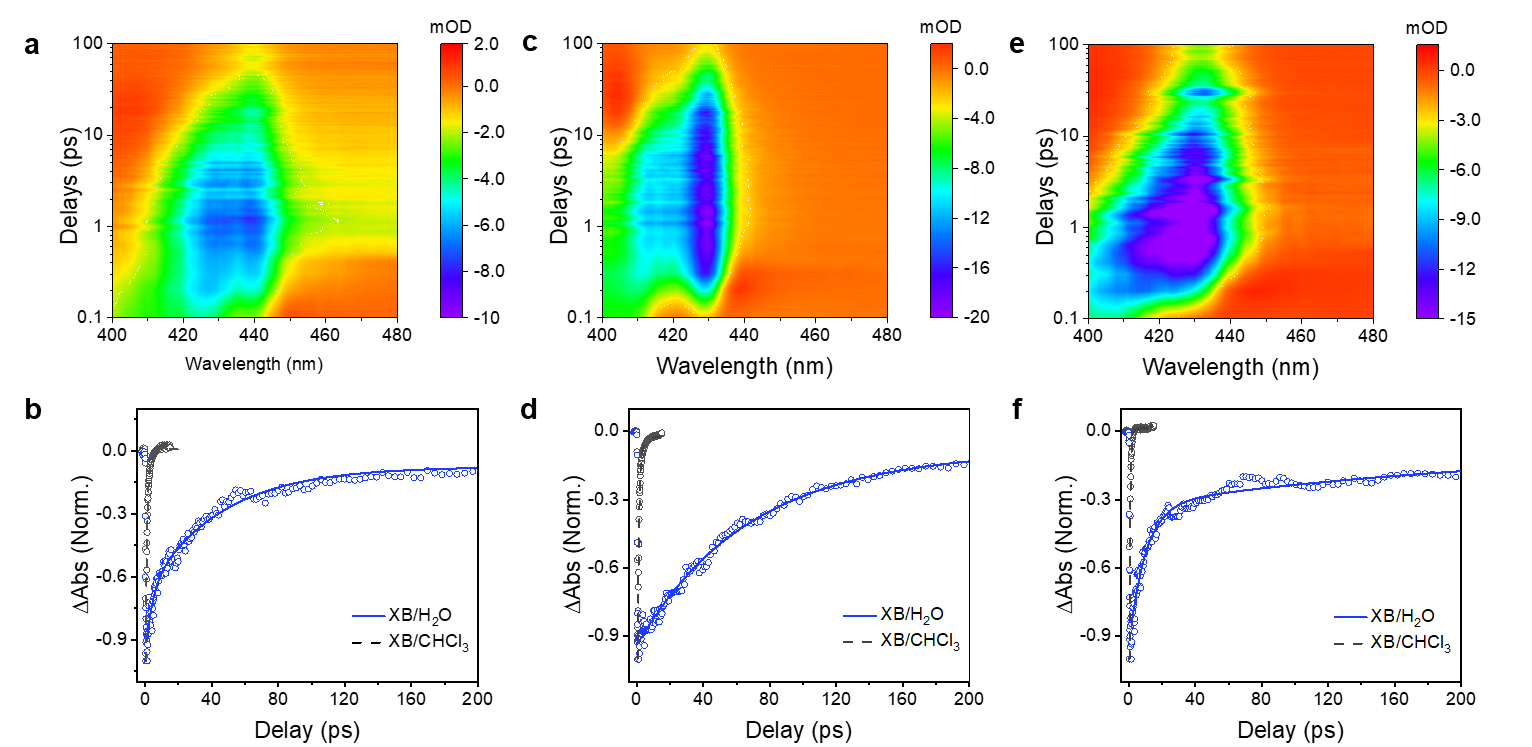
**

**Supplementary Fig. 23.** Transient absorption (TA) spectra of ZnSe QWs in water after MPA passivation. Pseudocolour plots of the TA spectra pumped at 375 nm for (a) thick-short, (c) thin-160, and (e) thin-ultralongZnSe QWs, respectively. The broad PIA signals related with trapped electrons disappeared in all samples. TA kinetics of XB signals for (b) thick-short, (d) thin-160, and (f) thin-ultralong ZnSe QWs in CHCl_3_ and H_2_O. The lifetime of XB in all samples enhances by orders of magnitude after MPA passivation, a consequence we attribute to the fact that the strong electron-donating ability of thiol group in MPA ligands enables the elimination of surface electron traps in ZnSe QWs.

**Supplementary Tables**

**Supplementary Table 1.** The fitting positions and corresponding FWHM of 1S_e_-1S_h_ exciton features of one-dimensional ZnSe nanocrystals with different sizes.

| **Size**  **(D**×**L, nm)** | **First exciton transition position (nm)** | **FWHM (nm)** | **Ref.** |
| --- | --- | --- | --- |
| 2.8×8.0 | 384 | 23.98 | [2] |
| 2.4×90 | 345 | 35.96 | [3]  [3]  [3] |
| 3.5×52 | 393 | 33.80 |  |
| 4.2×40 | 404 | 31.46 |  |
| 4×30 | 410 | 21.03 | [4] |
| 1.2×50~200 | 304 | 36.09 | [5] |
| 2.8×20 | 380 | 50.97 | [5] |
| 2.8×10 | 382 | 41.67 | [5] |
| D: 2.4 | 377 | 14.59 | [6] |
| D: 3.6 | 411 | 22.05 | [6] |
| 2.4×80 | 345 | 58.77 | [7] |
| 6.3×69 | 368 | 52.49 | [7] |
| D: 8 | 430 | 54.54 | [8] |
| Aspect ratio of 3 | 396 | 17.26 | [9] |
| D: 6 | 414 | 23.52 | [9] |
| 4.5×85 | 402 | 34 | [10] |
| Thin-100 | 431 | 12.97 | This work |
| Thin-160 | 431 | 13.53 | This work |

Note that only ZnSe nanorods or nanowires with obvious 1S_e_-1S_h_ exciton peak are listed here.

**Supplementary Table 2.** Quantitative analyses on molar ratios of Zn and Ag in ZnSe nanowires after the removal of Ag_2_Se tips.

| **Element**  **Sample** | **Zn: Ag (molar ratio)** | **Zn: Ag (molar ratio)** |
| --- | --- | --- |
|  | **ICP-AES** | **EDS** |
| Thick-short | 1: 0.009 | 1: 0.008 |
| Thin-ultralong | 1: 0.004 | 1: 0.000 |

**Supplementary Table 3.** Element ratios in different ZnSe QWs.

| **Element**  **Sample** | **Zn: Se (molar ratio)** | **Zn: Se (molar ratio)** |
| --- | --- | --- |
|  | **ICP-AES** | **EDS** |
| Thick-short | 1: 1.10 | 49.47: 50.53 |
| Thin-100 | 1: 0.89 | 48.58: 51.72 |
| Thin-160 | 1: 1.04 | 49.00: 51.00 |
| Thin-ultralong | 1: 0.96 | 49.11: 50.89 |

**Supplementary Table 4.** Synthetic parameters for different ZnSe QWs.

| **Size**  **(D**×**L, nm)** | **Ag_2_Se**  **reaction solution (mL)** | **Ripening time**  **(min)** | **Zn(Ac)_2_**  **(mg)** | **Se precursors** | **Reaction time**  **(min)** |
| --- | --- | --- | --- | --- | --- |
| Thick-short  8.5±1.1×45±10 | 3 | 8 | 40 | 9 mL  Se-OAm | 3 |
| Thick-short  7.4±0.7×45±5 |  | 4 |  |  | 1/6 |
| Thin-100  4.5±0.3×95±11 | 3 | 0 | 40 | 9 mL  Se-OAm | 3 |
| Thin-55  4.5×55±5 |  | 0 |  |  | 1/6 |
| Thin-45  6.5±0.6×44±6 |  | 4 |  |  | 1/6 |
| Thin-160  4.9±0.4×159±23 | 1 | 0 | 60 | 9 mL  Se-OAm | 3 |
| Thin-ultralong  5.9±0.7×μm | 1.5 | 0 | 120 | 44.4 mg SeO_2_ | 10 |

**Supplementary Table 5.** Comparison of the photocatalytic H_2_ production performances for ZnSe nanocrystal photocatalysts.

| **Photocatalyst** | **Amount (mg)** | **Sacrificial agents** | **Light** | **H^2^ production rate**  **(mmol·h^-1^·g^-1^)** | **Temp.** | **Ref.** |
| --- | --- | --- | --- | --- | --- | --- |
| ZnSe NPs^[a]^  ca. 5 nm | 200 | Na^2^SO^4^ | UV | 0.02 | —— | [11] |
| ZnSe NPs  ca. 6.6 nm | 120 | Lactic acid | >420 nm | 0.15 | —— | [12] |
| ZnSe NPs | 25 | Na^2^S  Na^2^SO^3^ | >400 nm | 1.8 | —— | [13] |
| ZnSe NPs  20-25 nm | 360 | —— | Visible light | 0.04 | R. T. | [14] |
| ZnSe NRs  5.2 nm×30.0 nm | 0.1 | Ascorbic acid | >400 nm, AM 1.5G | 33.6±2.0  76.9±9.5 | 25 ^o^C | [15] |
| ZnSe-MPA | 0.1 | Ascorbic acid | >400 nm, AM 1.5G | 45.9±1.4 | 25 ^o^C | [15] |
| ZnSe-DETA^[b]^ Nanobelts | 50 | Na^2^S  Na^2^SO^3^ | >420 nm, AM 1.5G | 0.1 | 0.5 ^o^C | [16] |
| ZnSe NRs  2.1 nm×24.8 nm | 5-10 | methanol | >400 nm | 0.05 | R. T. | [17] |
| Single Au-tipped ZnSe NRs | 5-10 | methanol | >400 nm | 0.4 | R. T. | [17] |
| Double Au-tipped ZnSe NRs | 5-10 | methanol | >400 nm | 0.3 | R. T. | [17] |
| Thick-short  ZnSe QWs | 10 | Na^2^S  Na^2^SO^3^ | full-spectrum | 3.94 | 25 ^o^C | This work |
| Thin-100  ZnSe QWs | 10 | Na^2^S  Na^2^SO^3^ | Full-spectrum | 5.66 | 25 ^o^C | This work |
| Thin-ultralong  ZnSe QWs | 10 | Na^2^S  Na^2^SO^3^ | Full-spectrum | 9.9 | 25℃ | This work |

[a] NPs: nanoparticles. [b] diethylenetriamine.

**Supplementary References**

1. Klimov VI. Spectral and Dynamical Properties of Multiexcitons in Semiconductor Nanocrystals. *Annu Rev Phys Chem* 2007; **58**: 635-73.

2. Ning J, Kershaw SV, Rogach AL. Synthesis of Anisotropic ZnSe Nanorods with Zinc Blende Crystal Structure. *Angew Chem Int Ed* 2020; **59**: 5385-91.

3. Jia G, Banin U. A General Strategy for Synthesizing Colloidal Semiconductor Zinc Chalcogenide Quantum Rods. *J Am Chem Soc* 2014; **136**: 11121-7.

4. Ji B, Panfil YE, Waiskopf N*, et al.* Strain-controlled shell morphology on quantum rods. *Nat Commun* 2019; **10**: 2.

5. Ning J, Kershaw SV, Rogach AL. Temperature-Controlled Fragmentation and Ripening: Synthesis of ZnSe Nanorods with Variable Dimensions and Crystal Structure Starting from Ultrathin ZnSe Nanowires. *Chem Mater* 2020; **32**: 3960-9.

6. Ning J, Liu J, Levi-Kalisman Y*, et al.* Controlling Anisotropic Growth of Colloidal ZnSe Nanostructures. *J Am Chem Soc* 2018; **140**: 14627-37.

7. Chen D, Zhang H, Li Y*, et al.* Spontaneous Formation of Noble- and Heavy-Metal-Free Alloyed Semiconductor Quantum Rods for Efficient Photocatalysis. *Adv Mater* 2018; **30**: 1803351.

8. Li X, Zhang Y, Zhai L*, et al.* Rational Synthesis of 1D Hyperbranched Heterostructures with Enhanced Optoelectronic Performance. *Angew Chem Int Ed* 2020; **59**:1-7.

9. Cozzoli PD, Manna L, Curri ML*, et al.* Shape and Phase Control of Colloidal ZnSe Nanocrystals. *Chem Mater* 2005; **17**: 1296-306.

10. Li D, Xing G, Tang S*, et al.* Ultrathin ZnSe nanowires: one-pot synthesis via a heat-triggered precursor slow releasing route, controllable Mn doping and application in UV and near-visible light detection. *Nanoscale* 2017; **9**: 15044-55.

11. Sanchez-Martinez A, Ortiz-Beas JP, Huerta-Flores AM*, et al.* ZnSe nanoparticles prepared by coprecipitation method for photocatalytic applications. *Mater Lett* 2021; **282**: 128702.

12. Lopes PAL, Maia DLD, Silva, LA. Chalcogenide nanoparticles like Cd_x_Zn_(1-x)_S_y_Se_(1-y)_ applied to photocatalytic hydrogen production from natural seawater under visible light irradiation. *Mater Today Commun* 2020; **25**: 101503.

13. Dai FX, Zhao RY, Huai XD*, et al.* Magnetic ZnFe_2_O_4_@ZnSe hollow nanospheres for photocatalytic hydrogen production application. *Composites Part B-Engineering* 2019; **173**: 106891.

14. Mkhalid IA. Improved photocatalytic performance in Bi_2_S_3_-ZnSe nanocomposites for hydrogen production. *Ceram Inter* 2018; **44**: 22198-204.

15. Kuehnel MF, Creissen CE, Sahm CD*, et al.* ZnSe Nanorods as Visible-Light Absorbers for Photocatalytic and Photoelectrochemical H_2_ Evolution in Water. *Angew Chem Int Ed* 2019; **58**: 5059-63.

16. Qiu B, Zhu Q, Xing M*, et al.* A robust and efficient catalyst of Cd_x_Zn_1−x_Se motivated by CoP for photocatalytic hydrogen evolution under sunlight irradiation. *Chem Commun* 2017; **53**: 897-900.

17. Chen W, Li XJ, Wang F*, et al.* Nonepitaxial Gold-Tipped ZnSe Hybrid Nanorods for Efficient Photocatalytic Hydrogen Production. *Small* 2020; **16**: 1902231.
